# Workplace cafeteria and other multicomponent interventions to promote healthy eating among adults: A systematic review

**DOI:** 10.1016/j.pmedr.2021.101333

**Published:** 2021-02-23

**Authors:** Ashika Naicker, Archana Shrestha, Chandni Joshi, Walter Willett, Donna Spiegelman

**Affiliations:** a Department of Food and Nutrition, Durban University of Technology, Durban, South Africa; b Department of Nutrition, Harvard TH Chan School of Public Health, Boston, USA; c Center for Methods on Implementation and Prevention Science (CMIPS) Yale School of Public Health, New Haven, USA; d School of Medicine, Tufts University, Boston, USA

**Keywords:** Cafeteria, Workplace, Environmental intervention

## Abstract

The objective of this review is to evaluate evidence for the effectiveness of workplace cafeteria and other supporting multicomponent interventions to promote healthy eating and reductions in health risks among adults. We conducted an electronic search in EMBASE, CINAHL, EconLit, Ovid, Cochrane, Web of Science and PubMed for English-language articles published from 1985 to July 2019. Studies were original articles reporting the results of workplace cafeteria interventions to promote healthy eating and reduction in health risks. Outcomes were classified as changes in fruit and vegetable intake, health risk indicators, dietary intake, and food sales. Interventions were categorized as interventions targeting food quality or quantity, targeting price, targeting food choice at point of purchase, targeting improved supply, targeting client’s information, education or motivation and targeting organization policies. Behavioral change conditions used in interventions were identified using the COM-B system of behavioral change. Results were presented in a narrative summary. A total of 55 studies out of 6285 articles were identified for this review. Several studies used multicomponent interventions and the most featured interventions included interventions targeting food quality or quantity, targeting client’s information, education or motivation and targeting food choice at point of purchase. There is evidence that workplace cafeteria and other supporting multicomponent interventions resulted in higher intake of fruit and vegetables, improved dietary intake, improved health outcomes and healthy food sales. The findings of this review have the potential to inform future cafeteria-based and other supporting multicomponent workplace health interventions.

The review protocol was not registered in a repository.

## 1 Introduction

More than 39% of the world’s population is classified as overweight and 13% as obese (World Health Organisation, 2018). Obesity increases the risk of developing multiple diseases including cardiovascular disease, hypertension, dyslipidemia, type 2 diabetes, stroke, osteoarthritis, and some cancers (Afshin et al., 2017, World Health Organisation, 2018) Prevention of obesity is an international public health priority, given the critical influence of obesity on health and well-being. In 2015, excess body weight contributed to 4.0 million deaths and 120 million cases of disability-adjusted life-years among adults globally (Collaborators et al., 2017). In addition, the associated health care costs of obesity is on the rise. In 2014 in the US, the average spending attributed to obesity was $1901 per single obese individual, accounting for $149.4 billion nationally (Kim and Basu, 2016).

The food environment, incorporating the availability, accessibility, cost, quality and promotion of certain types of food, is a major determinant of dietary intake (Glanz et al., 2005). An unhealthy food environment contributes to unhealthy eating patterns (Elbel, 2011, Marteau et al., 2012, Schwartz et al., 2012). The modification of the food environment has the potential to promote and encourage healthy actions and can be used as a basis of workplace health interventions (Engbers et al., 2005). Workplaces are sedentary settings and places where energy-dense foods and beverages are commonly available (Anderson et al., 2009). From the economic lens, there is a growing concern about the economic burden of obesity in the workplace, induced by costs associated with absenteeism, sick leave, disability, injuries, and healthcare claims (Popkin et al., 2006). Nonetheless, on the positive side, the worksite provides a strategic setting for implementing programs to promote healthy eating, since employees spend up to 60% of their waking hours at the worksite (Engbers et al., 2005). The worksite can thus reach a large proportion of adults, including those unlikely to engage in a preventive health behavior program (Gorman et al., 2013). However, several factors could impede workplace health promotion initiatives, including worksite readiness, and intervention implementation (Wolfenden et al., 2018). Several systematic reviews have been conducted that evaluate the effectiveness of worksite health promotion trials (Engbers et al., 2005, Geaney et al., 2013, Ni Mhurchu et al., 2010). However, results from one review found that there are few studies that focused on the impact of food environmental modifications on dietary intakes and that the few studies containing an environmental component obtained inconclusive results (Anderson et al., 2009). Besides, it proves challenging to filter out successful intervention components in changing dietary behaviour (Schliemann and Woodside, 2019).

With a high proportion of adults around the world working in the formal workplace setting, it is of great interest to examine the food environment in the workplace to inform the development of health promotion initiatives. Hence, this systematic literature review aims to identify and assess the effectiveness of workplace cafeteria and other supporting multicomponent interventions to promote healthy eating. This review is positioned differently from other reviews as it reports outcome measures to improve healthy eating at worksites; changes in fruit and vegetable intake, health risk indicators, diet and food sales. Moreover, it catalogues interventions and sub-interventions as cafeteria and supporting non-cafeteria interventions and identifies behavioral components within interventions for translation into intervention success.

## 2 Methods

### 2.1 Search strategy and procedures

We used the preferred reporting items for systematic reviews and meta-analyses (PRISMA Checklist) to guide this systematic review (S1 File) (Moher et al., 2015). We searched multiple databases including EMBASE (general medicine), CINAHL (nursing & allied health), EconLit, Ovid, Cochrane, Web of Science and PubMed from 1985 to July 2019. MeSH search terms included: (1) Setting-based: cafeteria, canteen, school, workplace, worksite, campus, industry; (2) Intervention-based: nutrition, diet, dietary intervention, health promotion, primary prevention, health behaviour, health education, food, program evaluation (S2 File). We searched the citations of sentinel papers for additional sources. We included peer reviewed intervention studies published in English. Inclusion criteria were: (a) targeting adult employees aged ≥18 years; (b) non-drug and non-surgical interventions aimed at modifying the food environment and (c) intervention delivered at a workplace cafeteria (front and/or back of house), including studies with non-cafeteria interventions implemented out of the cafeteria space. We excluded interventions involving (a) vending machines, kitchenettes or food trucks, (b) studies that reported results of hospital staff, patients and visitors, and university staff and students collectively, (c) interventions focused on eating disorders, (d) intervention studies that evaluated commercial weight-loss programs or products, (e) studies only involving the delivery of nutritional advice/education to employees, (f) pharmacological (drug-based studies) and clinically based interventions, observational and modelling (analytical methodology) studies aimed at improving health outcomes of participants. Studies had to report the effect of workplace cafeteria interventions and other supporting multicomponent interventions on changes in (a) fruit and vegetable intake, (b) health risk indicators (body mass index (BMI), blood pressure, serum cholesterol levels, blood glucose levels), (c) dietary intake (macro or micronutrient) or (d) food sales, such as the sales of healthy food. Interventions were catalogued as interventions targeting food quality or quantity, targeting price, targeting food choice at point of purchase, targeting improved supply, targeting client’s information, education or motivation and targeting organization policies (S3 File). Using the COM-B system of behavior change (Michie et al., 2011), essential conditions for behavioral change: capability, opportunity and motivation were identified in interventions to translate intervention success (Table 3 S5 File). The methodological heterogeneity of the studies precluded meta-analysis and subsequently, a narrative summary of each study’s characteristics and findings is presented. We imported all papers (title and abstracts) into an endnote database and removed duplicates. Two researchers (AN, CJ) screened the titles and abstracts, and full paper if necessary, separately and independently using a screening verification checklist. Any disagreements and unsure studies regarding inclusion were resolved by discussion with the third researcher (AS) until consensus was reached.

### 2.2 Data extraction

A copy of the full text of papers were obtained for each of the included studies. The screening checklist was re-applied in assessing the content of the paper. Studies not meeting the review inclusion criteria were excluded; however, studies meeting the inclusion criteria and belonging to the same trial was included. Two reviewers in parallel, (AN and CJ) independently extracted information from all 55 studies using the Data Abstraction Form published by the Guide to Community Preventive Services (Zaza et al., 2000) to classify and describe key characteristics of the intervention. The Guide to Community Preventive Services data collection instrument and procedure for systematic reviews balances the flexibility for evaluating papers with different study designs and intervention types with the need to ask specific questions to maximize validity and reliability, providing a structured format for reviewing paper content and quality (Zaza et al., 2000). A third reviewer (AS) double-checked 20% of the extracted studies for accuracy of data extraction. Thereafter, extraction results were compared for agreement and differences regarding data extraction were resolved by discussion until consensus was reached by all reviewers.

### 2.3 Quality assessment

Quality of study execution included an evaluation of five categories of threats to validity; study population and intervention descriptions, sampling, exposure and outcome measurement, data analysis, interpretation of results and other biases (S4 File), based on the Guide to Community Preventive Services guide which allows for the evaluation of different study designs with questions to evaluate a general concept. (Zaza et al., 2000). All studies that met the inclusion criteria were assessed by the two reviewers independently for their methodologic quality (S6 File). The reviewers scored the item as positive (+) if the item was met, negative (−) if the item was not met, and unclear (?) if insufficient information was provided. The total quality score was calculated by counting the number of items scored positively. Studies with none or one limitation were classified as good, 2–4 limitations as fair and 5 or more limitations as limited (Briss et al., 2000). Results were compared for agreement and differences regarding the quality score were resolved by discussion until consensus was reached by the reviewers.

## 3 Results

### 3.1 Study selection

**Fig. 1:**
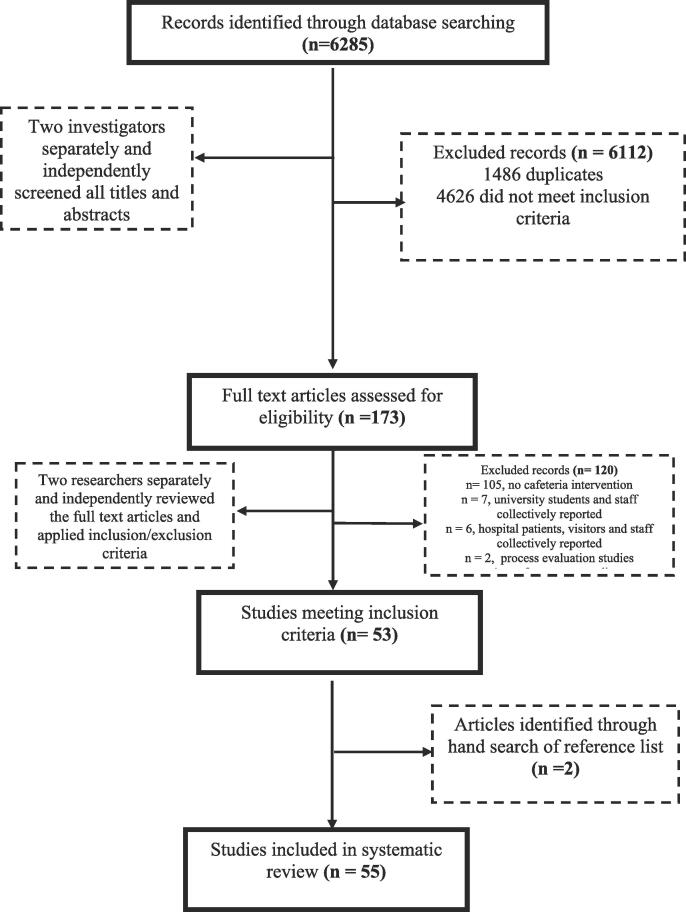
Flow chart of study selection.

The PRIMSA diagram showing the literature search and selection process is presented in Fig. 1. Electronic database searches generated 6285 potentially relevant references. After screening the title and abstract, 6112 articles were excluded; 1486 duplicates and 4626 did not meet the inclusion criteria. Following the screening of the full text, 120 articles further did not meet the inclusion criteria. We added two hand searched articles (Iriyama and Murayama, 2014, Uglem et al., 2013). A total of 55 articles conducted from 1994 to July 2019 were retrieved for detailed evaluation.

### 3.2 Study characteristics

Table 1 summarizes the study characteristics of the 55 papers reviewed. The papers included a range of different study designs; 23 randomized controlled trials, 13 non-randomized trails 14 pretest–posttest design and 5 time series. Many papers reported on more than one outcome. Papers classified by outcome yielded 17 papers on changes in fruit and vegetable intake, 16 papers on changes in health risk indicators, 21 papers on changes in dietary intake and 24 papers on changes in food sales. The duration of the intervention delivery ranged from 3 weeks to 5 years. Studies were conducted across multiple countries; 26 studies were conducted in the USA, 19 in Europe, 3 Japan, 2 in Brazil; and one each in Chile, Mexico, New Zealand, Australia and Taiwan. Studies were conducted in the private and public workplace settings ranging from government, factory, manufacturing, research, military, shipping, hospital, finance, farm, sports club, university and education. Eight studies took place in multiple worksites, and 15 studies did not indicate the worksite type. The study sample sizes ranged from 26 to 5695 employees and education level, ethnicity and male and female ratio varied among studies. Twenty-four studies used a single component intervention strategy, while 31 studies used multicomponent intervention strategies. Thirty-three studies used interventions targeting food quality or quantity, 12 studies used interventions targeting price, 24 studies used interventions targeting food choice at point of purchase, 5 studies used interventions targeting improved supply, 31 studies used interventions targeting client’s information, education or motivation, and 5 studies used interventions targeting organizational policies.

**Table 1:** Characteristics of studies implementing workplace cafeteria and other multicomponent interventions at worksites.

Study attribute	n (%)
Study design	
RCT	23 (42)
Non-randomized control trail	13 (24)
Time series	5 (9)
Pre/Post	14 (25)

Outcomes of interest	
Changes in fruit and vegetable intake	18
Changes in health risk indicators	16
Changes in dietary intake	20
Changes to food sales	24

Primary Location	
USA	26 (47)
Europe	19 (35)
Japan	3 (5)
Brazil	2 (3)
Chile	1 (2)
Mexico	1 (2)
Taiwan	1 (2)
Australia	1 (2)
New Zealand	1 (2)

Workplace type	
Multiple	8(15)
Government	7(13)
Factory	1(2)
Manufacturing	4(7)
Research	2(2)
Military	1(2)
Shipping	1(2)
Hospital	12(22)
Finance	1(2)
Farm	1(2)
Sports club	1(2)
University	1(2)
Not indicated	15(27)

Gender	
Men	5 (9)
Women	0 (0)
Both men and women	50(91)
Race and ethnicity	7(13)

Intervention	
Single component	24(45)
Multicomponent	31(55)
Interventions targeting food quality or quantity	33
Interventions targeting price	12
Interventions targeting food choice at point of purchase	24
Interventions targeting client’s information, education or motivation	31
Interventions targeting organizational policies	5

### 3.3 Effect of interventions to promote healthy eating and reduced health risks

Table 2 and Table 3 S5 File provides a summary of studies reporting changes in fruit and vegetable intake, health risk indicators, dietary intake and food sales as outcomes.

**Table 2:** Summary of studies reporting changes in fruit and vegetable consumption, changes in health risk indicators, changes dietary intake and changes to food sale as outcomes of workplace cafeteria and other multicomponent interventions.

Reference/ Author, year, country	Study design, setting and participants	Intervention and intervention duration ✓Cafeteria ✓✓Non-cafeteria ✓✓✓Both	Intervention comparator	Outcome				Quality
				Changes in FV consumption	Changes in health risk indicators	Changes in dietary intake	Changes in food sales	
(Bandoni et al., 2011), Location: São Paulo, Brazil	Group randomized trial. 29 worksite cafeterias enrolled in the Workers’ Food Program offering subsidized meals. Cafeterias had to prepare and distribute at least 150 meals daily. BL: n = 1296 (IG: 651, CG: 645), F/U: n = 1214 (IG: 630, CG: 584). Female: BL 59.6% (IG), 31.4% (CG); F/U 32.9% (IG), 32.7% (CG).	✓*Targeting organizational policies:* Production of a manual for cafeteria managers. ✓*Targeting improved supply*: Culinary workshops for cafeteria workers. ✓*Targeting client’s information, education or motivation:* Educational materials distributed at cafeteria to encourage FV consumption and poster displays to summarize the main points of the previous intervention strategies.Duration: 6 months.	No intervention in CG. CG received copies of education material at the end of the intervention.	-FV intake higher by 11.75 g/day in the meals (95% CI: 2.73, 20.77; P < 0.05).-Mean FV intake at F/U was 123.03 g/day (95%: CI 117.14, 128.93; P < 0.05).Effect size: Mild	n/a	-Total fat reduced by 4.27% in the IG (95% CI: 10.20, 1.66; P < 0.05).-Fiber increased by 1.35 g in meals at F/U (95% CI: 062, 3.33; P < 0.05).	n/a	Fair
(Beresford et al., 2001), Location: Seattle, USA	Randomized trial. Blue and white-collar workers from 28 worksites (14: IG, 14: CG) with a staff complement of 250 to 2000 employees from hospitals, educational, government, professional agencies, construction, manufacturing, financial institutions, retail, wholesale and service organizations. n = 1428 (IG), n = 1400 (CG). Female: 59.1% (IG), 57% (CG).	**✓***Targeting organization policies: An* employee advisory board was set up to support changes at worksites. **✓***Targeting food quality:* Provisions of more FV as part of the regular menus. **✓✓✓***Targeting client’s information, education or motivation:* Worksite wide educational opportunities (taste tests, cooking demonstrations). Duration: 2 years.	No intervention in CG.	-FV intake higher by 0.3 daily servings in IG compared to CG (P < 0.05).Effect size: Mild	n/a	n/a	n/a	Good
(Engbers et al., 2006), Location: Hague, Netherlands	Non-randomized trial. 2 government worksites with 4400 office employees. Participants must be able to climb stairs, have a BMI ≤ 23 kg/m^2^ and a contract of at least the duration of the intervention. BL: n = 244 (IG), 271 (CG); 3 months: n = 217 (IG), 245 (CG); 12 months: n = 191(IG), 241 (CG). Mean age: (BL) 45.3 years (IG), 45.5 years (CG). Female (BL): 36.9% (IG), 42.1% (CG).	**✓***Targeting food choice at point of purchase:* Placement of informational sheets near food products with caloric value translated into the number of minutes to perform a certain activity. **✓***Targeting client’s information, education or motivation:* An information stand was placed in the canteen with brochures and leaflets on healthy food, blood pressure and cholesterol. **✓***Targeting food quality*: Every 2 months for 1 day a week a healthy buffet was offered. Duration: 12 months.	No intervention in CG.	–No effects were found on FV intake.Effect size: Mild	n/a	–No effects were found on fat intake.	n/a	Limited
(Franco et al., 2013), Location: Rio de Janeiro, Brazil	Pre/Post. A scientific food technology research company with 130 employees including researchers, administrative assistants, general workers and sub-contracted staff. Workers who ate lunch in the company cafeteria on the 3 days of data collection were included in the study. n = 61 (both surveys). Mean age: 40 years. Female: 42.6%.	**✓***Targeting price:* Workers were provided with a meal voucher. Fruit and desserts were sold at a fixed price. **✓***Targeting improved supply:* The canteen operator and the nutritionist was made more aware of the importance of promoting FV. The nutritionist created menus and supervised the production of meals. **✓***Targeting client’s information, education or motivation:* A food tasting stand was set up showcasing FV dishes. Table displays were set up to promote FV consumption. Duration: 9 months.	No intervention in CG.	-FV intake higher by 53.6 g/day from BL to F/U (p = 0.01).-An increase of 0.66 servings of FV.-Vegetables recorded a greater variation of 38.5 g/day (p = 0.003) compared to fruits (15.8 g/day, p = 0.27) from BL to F/U.Effect size: Mild	n/a	n/a	n/a	Fair
(Beresford et al., 2000), Location: Seattle, USA	Randomized trial. 28 worksites with blue and white-collar workers. 6 health service organizations; 8 educational, governmental, or professional agencies; 4 construction manufacturing groups; 2 financial institutions; 2 retail trade groups; 2 wholesale trade groups; and the remainder were service organizations. n = 3500 (IG: 1750, 125 dropouts = 1625, CG: 1750).	**✓***Targeting food quality:* Structural changes in food availability to provide more FV as part of their regular menus. **✓***Targeting organizational policies*: The formation of an employee advisory board at each work site. **✓✓✓***Targeting client’s information, education or motivation*: Regular message posting about 5-a-Day and worksite wide educational opportunities (taste tests and cooking demonstrations). Duration: 2 years.	No intervention in CG.	-FV intake higher by 0.5 servings/day among participants reading 4 types of information compared to participants reading no material (p = 0.05).Effect size: Mild	n/a	n/a	n/a	Good
(Thorsen et al., 2010), Location: Denmark	Pre/Post. 5 Danish worksites with in-house non-profit food service facilities. Selection criteria: recruited facilities should serve > 50 but < 500 meals per day; represent different working environments; represent diverse company employee groups with respect to sex distribution and occupation, from physically exerting to sedentary work; and to be led by managers who were motivated towards change.	**✓***Targeting improved supply*: An 8-hour training session for all canteen staff. Goal setting (average grams of total FV consumed per customer per meal) by canteen staff. Duration: 5 years.	n/a	-FV intake higher by 95 g per meal per day from BL to F/U (P < 0.001).Effect size: Moderate	n/a	n/a	n/a	Limited
(Buller et al., 1999), Location: Arizona, USA	Randomized trial. Blue-collar employees from 10 public employers from county and city governments, public universities, community colleges, and public-school districts. n = 2091 (BL), 905 (study cohort), 42 (peer educators). Mean age: 43% (BL), 42% (study cohort), and 40.67% (peer educators). Female: 26% (BL), 25% (study cohort), 29% (peer educators).	**✓✓✓***Targeting client’s information, education or motivation:* Five a Day Education Program using standard formal communication channels (e.g., workplace mail, cafeteria promotions and speakers). Duration: 18 months.	CG received a general five a day program.	-FV intake higher by 0.77 daily servings among IG compared to CG at 18 months (P < 0.001).Effect size: Moderate	n/a	n/a	n/a	Limited
(Kushida and Murayama, 2014), Location: Niigata, Japan	Non-randomized trial. 16 workplaces with cafeterias were assigned to IG (n = 8) or CG (n = 8). n = 349 (IG: 181, CG: 168) Japanese male workers who visited the cafeterias ≥ 3 times/week. Mean age: 40.6 (IG), 42.0 (CG).	**✓***Targeting client’s information, education or motivation:* At the IG sites, 12 types of informational table tents were placed once every 2 weeks on all tables in each cafeteria. Information about vegetable consumption was presented in stages. Duration: 24 weeks.	No intervention in CG however, after BL personalized feedback was provided from diet history questionnaire.	-Vegetable intake higher in the cafeteria by 0.18 servings in IG compared to CG (P = 0.01).-Vegetable intake higher by 0.32 servings per day among IG compared to CG (P = 0.01).Effect size: Mild	n/a	n/a	n/a	Limited
(Uglem et al., 2013), Location: Norway	Randomized trial. 2 military camps at the Norwegian National Guard. n = 976 (IG: 739 (BL), 374 (F/U), (CG: 237 (BL), 105 (F/U). Mean age: 19.7 years (IG), 19.2 years (CG); Male: 100%	**✓***Targeting food quality:* A self-service salad bar consisting of a large variety of vegetables was introduced for the lunch meal. For dinner, vegetables were included in newly developed dishes, or vegetables were offered as side dishes. Bread with a wholegrain content of 50–100%, and a fiber content of 4–7 g/100 g was offered at all meals. **✓***Targeting client’s information, education or motivation:* Information about the health benefits of a diet rich in FV and whole grain cereals were given to the recruits in an information meeting and through posters, brochures and folders. 3 different posters, 5 of each, were present at the same time, being replaced with new versions every 6 weeks, containing information about main health effects of vegetables and whole grain bread. Duration: 5 months.	No intervention in CG.	-An average daily increase of 82 g vegetable consumption from BL to F/U (p < 0.001).Effect size: Moderate	n/a	- An average daily increase of 47 g semi-whole grain bread consumption (p < 0.001) from BL and F/U in the IG.	n/a	Limited
(Leighton et al., 2009), Location: Santiago, Chile	Pre/Post. Metal mechanic company with 171 employees. Employees who had a 5 day a week lunch at the industry canteen not under treatment for diabetes mellitus, hypertension, blood hypertension and dyslipidaemia were included, excluding employees who followed a weight loss programme in the previous 6 months and undergoing pharmacological treatment with drugs that modify lipid profiles, blood pressure, carbohydrate metabolism, plasma antioxidant capacity and inflammation. Mean age: 39 years. 11% female initially studied but were excluded from the 12-month analysis. Males: 145 (BL), 96 (F/U).	**✓***Targeting food quality:* The food offer during the intervention period included a salad bar presented everyday with different mixed salads, plus 2 options for main dish and natural fruits as a dessert. To encourage salad consumption an olive oil-based salad dressing containing herbs and spice was continuously available. Mediterranean diet menu, a vegetarian dish was available plus an option of beef with rice for consumers not interested in adhering to the Mediterranean diet. An olive oil bottle was routinely available at the salad bar counter. Duration: 1 year.	n/a	-Increased average daily consumption of vegetables at lunch by 89 g per person, P < 0.001.- Increased average daily consumption of fruit at lunch by 59 g per person, P < 0.000.Effect size: Moderate	-WC lowered by 1.7 cm (P = 0.002).-SBP lowered by 13.2 mmHg (P = 0.001).-DBP lowered by 14.9 mmHg (P = 0.001).-HDL cholesterol increased + 0.89 mg/dl, P < 0.000.- Not significant decrease of blood glucose and plasma triglyceride.	-Mediterranean diet score increased from 4.8 to 7.4.	n/a	Limited
(Thorsteinsson et al., 1994), Location: Iceland	Randomized trial. Employees at Grundartangi ferro-alloy factory who had to eat at least one hot meal per day served at the factory kitchen. No participants that were pregnant or taking lipid-lowering drugs. n = 155 (38 dropouts) = 117. IG1 A = 43, IG2 B = 58, IG3 = 31, IG4 = 23. Mean age: IG1: 38.1 years, IG2: 43.4 years, IG3: 44.5 years, IG4: 45.5 years. Male: 100% (females dropped off due to pregnancies).	**✓***Targeting food quality:* Menu ingredient changes included whole milk replaced with skimmed milk, a bread spread with less fat and fiber rich bread. Fat content of the lunch meals was decreased, and vegetables and salads were added to the menu. Lunches were calculated at about 1000 kcal, breakfast about 700–800 kcal, and the bread and biscuits in the coffee breaks contained fewer calories. **✓✓***Targeting client’s information, education or motivation*: Included consultations, written instructions, additional blood lipid measurements and meetings with the dietitian. Duration: 2 years.	All groups received intervention. Two groups (C and D) with the highest cholesterol levels received more interventive attention.	n/a	-Mean serum cholesterol was lowered by − 8.28% (-0.55 mmol/l) for the whole group (p < 0.001) after two years.-In subgroups studied C and D, HDL increased (0 < 0.001).- No significant change in BMI.	n/a	n/a	Limited
(Geaney et al., 2016), Location: Cork, Ireland	Non-randomized control trial. 4 manufacturing worksites with > 250 employees. Only permanent, full-time employees who purchased and consumed at least 1 main meal from the workplace canteens daily were eligible. Employees were excluded if they did not work full-time, travelled regularly for work (≥once a month); were medically advised not to participate in the study; were on long-term sick leave, pregnant or were involved in an on-going diet program. CG = 111, IG1 = 226 (nutrition education), IG2 = 113 (environment dietary modification), IG3 = 400 (combined- education and environment dietary modification). Age range: 30–44 years (64%). Female: 24.0%.	**✓***Targeting food quality or quantity:* Reduction of saturated fat, sugar and salt, increase in fiber and FV, portion size control. **✓***Targeting price:* Price discounts for whole fresh fruit. **✓***Targeting food choice at point of purchase:* Strategic positioning of healthier alternatives. **✓✓***Targeting client’s information, education or motivation*: Nutrition education comprised of monthly group nutrition presentations, detailed group nutrition information (daily and monthly posters, leaflets and emails) and individual nutrition consultations. Each participant attended three individual nutrition consultations (BL, F/U at 3–4 months and follow-up at 7–9 months). Duration: 3–4 months.	No intervention in CG.	n/a	-BMI lowered by 1.2 kg/m^2^ (95% CI: −2.38, −0.018; p = 0.047) from BL to F/U in the combined IG.- No effect on diastolic, systolic blood pressure and waist circumference.	-Saturated fat reduced by −5.2 g/day (95% CI: −9.4, −1.1; p = 0.013) from BL to F/U in the combined IG compared to CG.-Salt reduced by −1.3 g/day (95% CI: −2.3, −0.3; p = 0.010) from BL to F/U in the combined IG compared to CG.-Nutrition knowledge score increased by + 4.2 (95% CI: 0.3, 8.2; p = 0.034) from BL to F/U in the combined IG compared to CG.	n/a	Fair
(Ferdowsian et al., 2010), Location: Maryland and Virginia, USA	Non-randomized trial. 2 corporate government employees’ insurance companies. Inclusion criteria included individuals aged 21–65 years with a BMI > 25 kg/m2 and/or previous diagnosis of type 2 diabetes. Exclusion criteria included a history of unresolved alcohol or drug abuse or dependency; pregnancy; history of severe mental illness; unstable medical status; current use of a low-fat, vegetarian diet; or a HbA1c > 10.5%. n = 113, (IG) 68, CG (45). Female 73.5% (IG), Female 95.56% (CG).	**✓***Targeting food quality:* Low-fat vegan options offered daily. **✓✓✓***Targeting client’s information, education or motivation:* Group meetings with presentations, group discussion and cooking demonstration. **✓✓***Other*: Daily multiple vitamin to meet vitamin B12 requirements and tracking weight. Duration: 22 weeks.	No intervention in CG.	n/a	-Mean weight decreased by 5.1 kg in IG compared to an increase of 0.100 g in the CG (p < 0.0001).-BMI decreased by 2.0 kg/m^2^ in IG.-Mean WC decreased 4.7 cm in IG compared to increase of 0.8 cm in CG (p < 0.0001).- SBP and DBP did not change in the IG.-LDL and HDL cholesterol decreased but not statistically significant.	-Decrease of 6.2%E from sat fat, 14.2%E from total fat and increase of fiber by 10.1 g in IG at 22 weeks (p < 0.0001).	n/a	Fair
(Goetzel et al., 2010), Location: USA	Non-randomized trial. 12 worksites some with cafeterias totaling 10,281 employees including laborers, clerical staff, technical workers, professionals, managers, sales and administrative staff. Health risk assessment cohort n = 2431, IG = 1902 (high intensity = 1520, moderate intensity = 382, CG = 529). Biometric screening: n = 1521 (high intensity = 926, moderate intensity = 213, CG = 382). Mean age: 43 years. Female: 25%.	**✓***Targeting food quality:* Changing cafeteria menus. **✓***Targeting food choice at point of purchase:* Point of choice messages to encourage healthy eating and physical activity by strategically placing signs in front of cafeterias. **✓✓***Targeting client’s information, education or motivation*: Health promotion and risk reduction programs. Dissemination of health education materials; physical activity and weight management counselling. Duration: 2 years.	No intervention in CG.	n/a	-Weight maintained in IG and increased by 1.3 lb in CG over 2 years (p < 0.01).-BMI maintained in IG and increased by 0.2 kg/m^2^ in CG over 2 years (p < 0.01).- SBP lowered by −7.0 mmHg after 2 years (p < 0.001).- DBP lowered by −1.6 mmHg after 2 years (p < 0.001).- Cholesterol lowered by −3.6 mg/dL after 2 years(P < 0.02).	n/a	n/a	Good
(Hjarnoe and Leppin, 2013), Location: Denmark, Greenland, Faroe Islands	Pre/Post. 2 Danish shipping companies with 630 employees (cargo company; 190 employees), (offshore rescue and support company; 440 employees). BL n = 606. F/U n = 362. Mean age: 42 years (BL), 44 years (F/U). Male: 100%.	**✓***Targeting improved supply*: Two-day course on healthy cooking for all chefs and staff with cooking responsibilities which was run over 5 alternate days. **✓✓***Other:* upgrading of fitness room facilities. Group based smoking cessation. Individual exercise guidance. Duration: 1 year.	n/a	n/a	-Metabolic syndrome lowered by 9% at F/U (p = 0.029).	-Intake of high sugar products reduced by 9% (P = 0.002).	n/a	Limited
(Fernandez et al., 2015) Location: North-Eastern, USA	Randomized trial. 10 non-unionized manufacturing, research, and development companies with 3799 blue and white-collar employees and some sites with cafeterias. Full time employee’s ≥ 18 years old. Mean age BL: 47.7 years (IG), 47.4 years (CG). Mean age F/U: 49 years (IG), 49.7 years (CG). Female BL: 31.8% (IG), 44.4% (CG); Female F/U: 41.2% (IG), 37.4% (CG).	**✓***Targeting food quality or quantity:* Low sodium soup and reducing meals by 100 calories. **✓***Targeting improved supply:* Chef training workshop and a refresher lead by the dietitian on ways to cook healthier. **✓***Targeting food choice at point of purchase*: Healthy beverage signs. **✓***Targeting price:* Half portions. FV sides subsidized using ‘Buy 3, Get 1 Free’ punch cards. **✓✓***Targeting client’s information, education or motivation:* Brochures on nutrition and physical activity. Educational posters and a website with wellness information. Duration: 2 years.	No intervention in CG.	n/a	-Mean BMI decreased by 0.54 kg/m^2^ (P = 0.02) in IG and 0.12 kg/m^2^ (P = 0.73) in CG; difference in differences decrease of 0.42 kg/m^2^ (P = 0.33).-Overweight or obese employees decreased by 3.7% (P = 0.07) in the IG and increased by 4.9% (P = 0.1) in CG resulting in a difference in differences decline of 8.6% (P = 0.02).	n/a	n/a	Limited
(Engbers et al., 2007), Location: Hague, Netherlands	Non-randomized trial. 2 government companies with 4400 employees. Employees must be able to climb stairs, BMI ≤ 23 kg/m^2^ and a contract of at least the duration of the intervention. Subjects who were pregnant or became pregnant during intervention year or had severe cardiovascular/musculoskeletal disorders were excluded. n = 694, IG = 333, CG = 361. Mean age: 45.3 years (IG), 45.5 years (CG). Female 37.4% (IG), female 41.7% (CG).	**✓***Targeting food choice at point of purchase:* Placement of informational sheets in close vicinity to food products. Every 4 weeks, 1 group out of 6 product groups was chosen and highlighted. Each group of food products was repeated once during the year. On the sheets the energy (kcal) value of 6 products was translated into the number of minutes needed to perform a certain activity to burn these calories. **✓✓***Other*: The stair use intervention consisted of placing point-of-decision prompts on elevator doors at the ground floor. Food steps were printed on the floor. Duration: 12 months.	No intervention in CG.	n/a	-Total cholesterol lowered by −0.35 mmol/l (95% CI: −0.55, −0.15; p < 0.01) in IG compared to CG for women at 12 months.-HDL increased by 0.10 mmol/l (95% CI: 0.06, 0.14; p < 0.01) in IG compared to CG for men at 12 months.-Increase of systolic blood pressure by 4 mmHg in the IG (P < 0.01).	n/a	n/a	Limited
(Mishra et al., 2013b), Location: USA	Randomized trial. 10 (5 IG, 5 CG) government employment insurance worksites. Employees had to be ≥ 18 years with a BMI of > 25 kg/m^2^ or a previous diagnosis of type 2 diabetes. BL: n = 291 (IG 142, CG 149). Mean age: 44.3 years (IG), 46.1 years (CG). Female: 77% (IG), 88% (CG). Ethnicity: non-Hispanic 89% (IG), 93% (CG). Occupation: service/ sales staff 63% (IG), 71% (CG).	**✓***Targeting food quality:* At intervention sites with cafeterias, food service managers were asked to include low-fat plant-based menu options, such as oatmeal, minestrone or lentil soup, veggie burgers and Portobello sandwiches, among the daily offerings. **✓✓***Targeting client’s information, education or motivation:* Weekly lunch hour classes and group discussion following an established curriculum. Duration: 18 weeks.	No intervention in CG.	n/a	-Mean body weight fell 2.9 kg in the IG (95% CI: −2.0, −3.9; P < 0.001).-BMI lowered 1.5 kg/m^2^, p < 0.001.-Total cholesterol fell 8.0 mg/dl (95% CI: −13.1, −2.9; P < 0.01).-LDL cholesterol reduced by −7.2 mg/dl (95% CI: −11.8, −2.7) in the IG compared to CG (P < 0.01).- HDL cholesterol reduced by −2.7 mg/dl (95% CI: −4.4, −1.1) in the IG compared to CG (P < 0.01).-HbA1c reduced by 0.6% point (95% CI: −0.29, −1.1; P < 0.05) in the IG compared to CG.	-% energy from total fat reduced by −15.2 (95% CI: –22.7, −7.6; P = 0.001).-% energy from saturated reduced by −6.7 (95% CI: −9.7, −3.7; P < 0.001).-Cholesterol lowered by −92 mg (95% CI: −141.5, −42.6; P < 0.001).- Fiber increased by 4.6 g (95% CI: 1.9, 7.2; P = 0.001).	n/a	Limited
(LaCaille et al., 2016), Location: Minnesota, USA	Non-randomized trial (Quasi experimental design). Mid-sized healthcare system. The IG consisted of employees from the hospital campus (including the main hospital, administrative offices, and several specialty outpatient clinics), whereas the CG consisted of employees from 6 primary care clinics. BL n = 407 (IG), 96 (CG).Mean age: 43.0 years. Female: 85.1%; White: 92.5%.	**✓***Targeting food quantity*: Changes included reducing the size of serving spoons (BL) and offering half portions at half price. **✓***Targeting food choice at point of purchase*: Food items in the hospital cafeteria was labeled with calories, number of steps required to burn those calories, and with a traffic light color rating. **✓✓✓***Targeting client’s information, education or motivation:* Messages were offered through posters, table toppers, and a website in 3 phases. In the first phase, messages focused on educating employees about the meaning of the “traffic light” labels. The goal of the second phase was to educate employees about the meaning of energy balance and portion sizes. The final phase focused on underscoring the role of social support in losing and maintaining weight-loss. Duration: 1 year.	No intervention in CG.	-FV servings did not significantly differ between groups over time, with the IG showing a significant decline over 12 months (-0.35 servings/day, p = 0.007).Effect size: Mild	- Neither group showed significant decrease in weight, BMI or WC.	n/a	n/a	Fair
(Brehm et al., 2011), Location: Kentucky, USA	Randomized trial. Eight (4 IG, 4 CG) small manufacturing companies ranging in size from 150 to 350 employees. Cafeteria intervention in 1 worksite. Participants were required to be ≥ 18 years of age. Pregnant and lactating women, those who were unable to speak or read English, and temporary workers were excluded from the study. n = 534. Mean age: 43.8 years (range of 19–72). Female: 40%.	**✓***Targeting food quality or quantity:* Taste tests with employees and researchers which lead to recommendations for improving the nutritional value of foods served in the cafeteria. Examples of recommendations included: (1) standardize and reduce portion sizes of entrees; (2) offer half portions of entrees; replace full-fat cheeses with reduced-fat cheeses on sandwiches and in recipes; (4) offer at least one healthier entrees on the menu; (5) offer a greater variety of fresh FV. **✓✓✓***Targeting organizational policies*: Employee advisory committees and walking paths. Duration: 1 year.	No intervention in CG.	n/a	–No significant differences were found between IG and CG in BMI, body fat, or key bio measures related to cardiovascular health.-Cholesterol lowered by −9.3 mg/dL, LDL lowered by −5.5 mg/dL, triglyceride lowered by −20.8 mg/dL, fasting glucose lowered by −1.5 mg/dL (P < 0.05)	-Lower intake of saturated fat and cholesterol in the IG compared to CG (p < 0.05).	n/a	Limited
(Linde et al., 2012), Location: USA	Randomized trial. 6 worksites in a US metropolitan area. Worksites were eligible if they had 250–1000 employees, presence of a food service, a building with at least 2 floors and minimal seasonal fluctuations ofemployees. Employees were eligible if they were employed at 50% time on-site during a daytime shift. n = 2700 (2428 were eligible). Mean age: 42.9 years, range 18–75; Female: 62.6%; White: 88.6%.	**✓***Targeting food quality or quantity:* Foods were classified as calorie smart for healthy portion sizes. **✓✓***Targeting client’s information, education or motivation*: Posters and signs relating to healthy eating and exercise were placed in stairwells to enhance the stair environment. Other: physical activity was recorded with an infrared beam on staircases to record stair traffic. Duration: 2 years.	No intervention in CG. At the last round of data collection CG was offered a DVD containing training intervention material.	n/a	–No differences between IG and CG in weight change over the 2-year study period.-Mean weight gain of 0.13 kg/m^2^ at IG sites (95% CI: −0.21, 0.46; p = 0.36).	n/a	n/a	Fair
(Iriyama and Murayama, 2014), Location: Japan	Randomized trial: 6 months cross over intervention. Male workers with or at risk of obesity were recruited for this study at 5 worksites, of whom 57 were analyzed (IG, n = 28, CG, n = 29. Mean age: 45.5 years (IG), 46.0 years (CG). Male: 100%.	**✓***Targeting food quality:* Provision of healthy cafeteria meals along with nutritional information defined as a meal containing 600–700 kcal of energy and ≥ 120 g of vegetables, with a fat/energy ratio of 20–25%) was served only to the IG at each worksite cafeteria (five days/week) for 6 months. The IG was instructed to consume these menus > 3 times per week. **✓✓✓***Targeting client’s information, education or motivation:* Health information was provided for 24 weeks using weekly nutrition notes placed on food trays and one 20-minute individual counselling and a series of four 20-minute health education sessions in a small-group setting. Duration: 6 months.	CG received the intervention 6 months after the study.	n/a	-Body weight reduced by 1.8 kg at F/U (P = 0.017).-BMI reduced by 0.8 kg/m^2^ at F/U (P = 0.017).	n/a	n/a	Limited
(Inoue et al., 2014), Location: Japan	Non- randomized trial. Middle aged men engaged in desk work. Commute by train or bus with no participation in exercise. IG n = 28; CG n = 7. Mean age: 47.2 years; Male: 100%.	**✓***Targeting food quality*: IG received a Japanese style lunch which provided balanced nutrition and sufficient vegetable consumption over the course of three months (600 kcal ≤ Energy < 650 kcal, Fat < 18 g, Cholesterol ≤ 100 mg, Fiber ≥ 8 g, Total vegetables ≥ 130 g, Sodium chloride equivalent ≤ 3.8 g). Duration: 3 months.	No intervention in CG.	- Vegetable intake increased by 118.7 g, p = 0.035.-Effect size: Moderate	-Serum cholesterol lowered by 12 mg/dL, p = 0.06.-LDL cholesterol lowered by 11 mg/dL, p = 0.010.-HDL cholesterol lowered by 2 mg/dL, p = 0.07.-SBP lowered by −5.6 mmHg, p = 0.023.-DBP lowered by −7.6 mmHg, p = 0.001.	-Energy intake lowered by 450 kcal, p = 0.042.-Fiber increased by 15.1 g, p = 0.047.	n/a	Limited
(Levin et al., 2010), Location: IG: Maryland, CG: Virginia, USA	Non-randomized trial. 2 corporate government worksites. Employees at least 18 years, with BMI ≥ 25 kg/m2 and /or pre-existing diagnosis of type 2 diabetes (fasting plasma blood glucose concentration ≥ 126 mg/dl on two occasions or a prior physician’s diagnosis of type 2 diabetes).n = 113 (IG 68, CG 45). Mean age: 46 years (IG), 42 years (CG); Female: 78% (IG), 96% (CG). Ethnicity: Non-Hispanic: 56(IG), 32(CG).	**✓***Targeting food quality*: IG cafeteria included low fat vegan menu options such as oatmeal, minestrone or lentil soup, veggie burgers and Portobello sandwiches. Approximately 1 breakfast item, and 4 lunch items (two entrees and side dishes) that met the diet guidelines were offered. **✓✓✓***Targeting client’s information, education or motivation*: Cooking demonstrations and nutrition education. Duration: 22 weeks.	No intervention in CG.	n/a	-Weight lowered by −5.3 kg (95% CI: −7.0, −3.5; P < 0.0001).-WC lowered by −5.5 cm (95% CI: −7.3, −3.7; P < 0.0001).	-Energy mean effect size of −262.5 kcal (95% CI: −469.3, −55.7; p = 0.01).-Total fat mean effect size of −16.5 g (95% CI: −20.4,−12.5; p < 0.0001).-Trans-fat mean effect size of −1.2 g (95% CI: −1.7, −0.6; p < 0.0001).-Saturated fat mean effect size of −7.2 g (95% CI: −8.9, −5.5; p < 0.0001).-Cholesterol mean effect size of −129.3 mg (95% CI: −168.2, −90.4; p < 0.0001).- Vitamin C mean effect + 29 mg (95% CI: 13.8, 44.1; p < 0.0001).-Fiber mean effect size of 8.9 g (95% CI: 6.2,11.7; p < 0.0001).	n/a	Limited
(Mishra et al., 2013a),Location: USA	Randomized trial.10 government employment insurance worksites (5 IG, 5 CG). Employees had to be ≥ 18 years with a BMI of > 25 kg/m^2^ or with a previous diagnosis of type 2 diabetes.BL n = 271 (IG 130, CG 141). F/U: n = 183 (IG 78, CG 105). Mean age: 42.2 years. Female: 84%. Ethnicity: non-Hispanic 81%.Occupation: service/ sales staff 68%.	**✓***Targeting food quality or quantity:* Participants at intervention sites were asked to follow a low-fat vegan diet consisting of whole grains, vegetables, legumes, and fruits, with no restriction on energy intake for 18 weeks. They were asked to avoid animal products and to minimize added oils, with a target of < 3 g of fat per serving. They were also encouraged to favor foods with a low glycaemic index.**✓✓***Other*: IG participants were asked to take a daily supplement of vitamin B12.Duration: 18 weeks.	No intervention in CG.	n/a	n/a	-% energy from fat reduced by −5.4, (95% CI: −9.8, −0.9; P = 0.02).-% energy from saturated reduced by −2.9, (95% CI: −4.7, −1.1; P = 0.006).-% energy from monounsaturated fats reduced by −2.2 (95% CI: −3.8, −0.6; P = 0.01).-Cholesterol lowered by −50.2 mg (95% CI: −83.6, −16.8; P = 0.009).-Fiber increased by 4.5 g (95% CI: 2.3, 6.7; P = 0.002.-% energy from carbohydrate increased by 8.6 (95% CI: − 3.2, 13.9; P = 0.006).	n/a	Limited
(Lassen et al., 2011),Location: Denmark	Randomized trial. 8 blue-collar worksites of which 5 had canteens (IG 5, CG 3). BL employee dietary survey (n = IG 102, CG 66). BL canteen survey (n = IG 48, CG 24), F/U (n = IG 48, CG 24). Pregnant women and individuals not expecting to be present at the worksite at F/U were excluded.	**✓***Targeting food quality:* Healthy canteen choices, free cold water, reduced soda and candy products.**✓***Targeting price*: Free fruit program.**✓***Targeting client’s information, education or motivation:* Information and dialogue-based initiatives, food workshop/taste demonstrations, informational material (e.g., nutrition quizzes, dinner mats, computer-based activities, leaflets), monthly news magazine, healthy lunchtime clubs.Duration: 6 months.	3 CG sites with minimum intervention; 2 sites free fruit program and all 3 sites monthly news magazine.	-Fruit intake increased by 55 g/d, (95% CI: 16, 94; P = 0.007).- FV increased by 95 g/10 MJ, (95% CI: 36, 154; P = 0.002).Effect size: Moderate	n/a	-Decrease in intake of fat (-2.2% E, 95% CI: −3.4, −1.0; P = 0.002) in IG.-Cake and sweets lowered by −18 g/10 MJ, 95% CI: −29, −7; P = 0.002) in IG.-Increase in intake of dietary fiber by 3 g/10 MJ, (95% CI: 2,5; P = 0.001) in IG.-Decrease in %E from fat by 11% E; P < 0.001 in IG.	n/a	Limited
(Cook et al., 2001),Location: South Auckland, New Zealand	Non-randomized trial. 2 manufacturing sites with a stable workforce. n = 253: 132(IG), 121 (CG). All male hourly paid blue-collar workers except those known by management to be leaving within one year. Mean age: 35.0 (IG), 42.9 (CG) Male: 100%. Ethnicity: Pacific 56.1 (IG), European 25.7 (IG)	**✓**Targeting food quality: Inclusion of low-fat options and water as a beverage. **✓***Targeting food choice at point of purchase*: Point of choice messages promoting FV. **✓***Targeting client’s information, education or motivation:* Nutrition displays in the cafeteria.Duration: 6 months..	No intervention in CG.	-Significant difference in the change of vegetable intake (p = 0.007) with an increase at both 6 (p = 0.002) and 12 months (p = 0.05) in the IG.- No effect in fruit intake.Effect size: Mild	- SBP lower by 5 mmHg at 6 months (p = 0.001).–No significant difference in change in weight, BMI and WC.	-There was a strong relationship of the intervention to change in mean fat score (p = 0.0003) with greater reduction at IG and both 6 (p < 0.0001) and 12 months (p = 0.005).	n/a	Limited
(Geaney et al., 2010),Location: Cork, Ireland	Non-randomized trial.Two public sector hospitals: one with the catering initiative (IG) and one without a specific catering initiative (CG).n = 100 (IG 50), (CG 50). Hospital staff age range 18–64 years were eligible for the study if they consumed at least one main meal in the hospital staff canteen daily. Female: 80% (IG), 74% (CG).	**✓***Targeting food quality:* Reduction of food high in salt, fat and sugar. High-salt products and processed meat were replaced with low-salt options. Fresh herbs, spices and garlic were introduced to develop flavor. Salt was removed in all cooking. In the canteen, salt was removed from the tables, but salt sachets were available at service. No sauces or accompaniments were added to any meals without the customer’s consent. Cooking methods with oil were limited. Desserts were fruit base.**✓***Targeting price:* Staff members were encouraged to consume extra salad and vegetables options at no extra cost.**✓***Targeting client’s information, education or motivation:* Nutrition information on salt reduction and a healthy diet was displayed in the canteen area.Duration: 2 years.	No intervention in CG.	n/a	n/a	-Total sugar reduced by 25.27 g (95% CI: 10.67, 39.87; P < 0.001).-Total fat reduced by 23.4 g (95% CI: 12.69, 34.2; P < 0.000).-Saturated fat reduced by 11.4 g (95% CI: 6.45, 16.39; P < 0.000).-Salt reduced by 1.04 g (95% CI: 0.21, 2.06; P < 0.046).	n/a	Limited
(Emmons et al., 1999), Location: USA	Randomized trial. 22 worksites with a cohort of 2055 participants. n = 2761 (BL).Mean Age: 42.0 (IG), 41.8 (CG). Female: 42.2 (IG), 47.9% (CG).White: 92.6%. Completed high school: 83.6% (IG), 81.0% (CG).	**✓***Targeting food choice at point of purchase*: At the point of purchase in cafeterias/vending machines the food labels met the Working Well Trial (WWT) criteria for fat or fiber.**✓***Targeting organization policy*: Catering policy to follow WWT guidelines.Duration: 2.5 years.	No intervention in CG.	-Fruit and vegetable consumption increased marginally by 7%, p < 0.06.Effect size: Mild	n/a	-Fiber consumption increased by 11%, P < 0.001 at the final assessment point in the IG.	n/a	Limited
(Lassen et al., 2014), Location: Denmark	Non– randomized trial. 2 hospital worksite canteens. Intervention canteen had to have the ambition to become one of the pioneers in achieving the keyhole certification in Denmark. Employees were excluded if they ate lunch outside the canteen. n = 270 (BL, 6 months and F/U), IG = 135, CG = 135. Mean age: 41 years. Female: 46%. Occupation: 41% medical doctors and health care personnel.	**✓***Targeting food quality:* For keyhole labelled meals, all recipes were modified and taste tests conducted to assess the acceptability of the modified foods. Food intake and edible plate waste measured through validated digital photographic method. The food was also weighed for intake estimation.**✓***Targeting price*: Fixed price was given to all menus at IG canteen and the CG canteen had buffet-by-weight meals.Duration: 6 weeks from BL for certification and 6 months to (F/U).	No intervention in CG.	-FV increased by 17 g/100 g (95% CI: 39, 58; P = 0.002).Effect size: Mild	n/a	-Mean decrease in energy density in the consumed meals by 154 kJ F/U (P < 0.001) at intervention canteen.-At end-point participants consumed on average 20 E% less fat compared to B/L (P < 0.001).	n/a	Limited
(Lassen et al., 2012), Location: Denmark	Pre/Post. A financial worksite offering canteen takeaway meals. Eligible for inclusion were healthy men and non-pregnant women aged ≥ 18 years expecting to be present for the 7-week study period. n = 27. Mean age: 40 years (range 27–52); Female: 52%.	**✓***Targeting food quality*: Healthy meals offered following recognized nutrition recommendations.**✓***Targeting price*: CTA meals were offered twice weekly. Participants received CTA meals free of charge for themselves and for their families. Duration: 7 weeks. *Comparator*: Non CTA meal days.		-FV intake increased by 129 g for CTA evening meals (95% CI: 49, 210; p = 0.002).-Vegetable intake increased by 109 g/d (95% CI: 62,155; p < 0.001).Effect size: Moderate	n/a	-Average energy density of consumed CTA meals was lower by 187 kJ/100 g (95% CI: −225, −149; p < 0.001).	n/a	Fair
(Lowe et al., 2010), Location: Philadelphia, USA	Randomized control trial. 2 hospital cafeterias. Male and female hospital or university employees between the ages of 21 and 65 years were eligible if they ate lunch in the hospital cafeteria at least twice a week. n = 96. Hospital A = 53, Hospital B = 43. Environmental Change (EC) = 49, Environmental Change Plus Energy Density Education and Incentives (EC-Plus) = 47.Mean age = 44.2 years. Female: 81.25%.	**✓✓✓***Targeting food quality, targeting food choice at point of purchase, targeting price, targeting client’s information, education or motivation:* Two conditions: 1). only environmental change (EC group) (i.e., the introduction of 10 new low-energy–density (ED) foods and provision of labels for all foods sold at lunch, which listed ED, calories, and macronutrient content or 2.) the environmental change plus pricing incentives (EC-Plus) (i.e., low-ED foods and education on low-ED eating delivered in four, 1-hour group sessions. Duration: 3 months (intervention).	EC group.	-Significant condition by time interaction on reported fruit intake (F(1,71) = 5.41, p < 0.05; η_p_^2^ = 0.07): EC-Plus group increased fruit intake (from 0.77 servings to 0.98 servings)Effect size: Moderate	n/a		Over BL (2 months) and intervention periods (3 months), both the EC and EC-Plus groups decreased the overall energy content of their lunch purchases (F(4,66) = 7.20, p < 0.001; η_p_^2^ = 0.30)	Limited
(Berkowitz et al., 2016), Location: Minnesota, USA	Time Series. Worksite employees and restaurant employees at two food service establishments serving lunch to 125–200 employees daily. n = 521 (BL), 603 (Intervention period).	**✓***Targeting food quantity*: Consumption and plate waste data were collected for 5 weeks before and 7 weeks after introduction of 5 reduced-size entrées in a worksite lunch cafeteria. Full-size entrées were available throughout the entire study periods. Worksite employees could choose from the entrée of the day, cold and hot sandwiches, soup or salad bar for their lunch meal.Duration: 7 weeks.	n/a	n/a	n/a	-Energy intake decreased by 310 kJ, fat intake lowered by 4.3 g, cholesterol lowered by 19 mg/dL, and sodium by 106 mg when both full and reduced sized entrees were offered (P < 0.0001).	-A small proportion of reduced sized entrees were selected (5.3–12.8%).	Limited
(Vermeer et al., 2011), Location: Amsterdam, Netherlands	Randomized trial. 25 worksite cafeterias with 308 participants from 15 hospitals, 5 companies, 3 universities and 2 police departments. Participants had to consume a hot meal at the worksite cafeteria at least once a week. IG 1 (n1 = 129), IG 2 (n2 = 75), CG (n = 104). Mean age: 39.18 years, range 18–79; Female: 50%; Education: Tertiary level 70.5%.	**✓***Targeting food quantity:* IG 1: smaller portion (2/3 the size of the existing portion) was offered in addition to the existing portion and proportional pricing. IG 2: smaller portion was added to the assortment and value size pricing (that is, a lower price per unit for large portions than for small portions). Duration: 3 months.	No intervention in CG.	n/a	n/a	n/a	–No effect of proportional pricing and value pricing was found *B* = -0.11 (0.33)*,* (CI: −0.76, 0.54; P = 0.74).	Fair
(Steenhuis et al., 2004),Location: Netherlands	Randomized trial. 17 Dutch companies and government organizations. Mainly white-collar workers visiting the worksite cafeteria. Cafeterias were selected if they had>400 cafeteria visitors per day and a range of food items that allowed an increase of at least 4 further food items as well as labelling. n = 1013, IG1 = 215, IG2 = 290, IG3 = 293, CG = 215. Mean age: 38 years, range 18–64 years; Female: 38%.	**✓✓✓***Targeting food quality, targeting client’s information, education or motivation, targeting food choice at point of purchase:* IG1: Food supply plus educational program (FSP) , IG 2: Labeling program plus educational program (LP), IG 3: Educational program (EP), CG: No program (NP). An increased availability of low-fat products and FV. Attention was drawn to the new added products by placing a sign in front of them with the phrase ‘new and healthy’ on it. In the labelling program, low-fat products in 6 food product categories were labelled with a sign in front of the product. The labelling was explained to the cafeteria visitors using posters and table tents. Information was given to increase awareness, change attitudes, increasing self-efficacy, teaching skills and managing social influences. Duration: 1 month, and it could be prolonged and supported up to 6 months.	CG: No program (NP).	n/a	n/a	–No significant effects on consumption data were found for any of the programs.	-Sales data revealed a significant effect of the labelling program on desserts, LP versus EP, p < 0.01; LP versus NP, p < 0.05.	Good
(Thorndike et al., 2014), Location: Massachusetts, USA	Time series. 1 large hospital cafeteria used by 2285 hospital employees and with mean transactions of 6511 daily. Mean age: 43 years; Female: 73%.	**✓***Targeting food choice at point of purchase*: Phase 1 was a 3-month color-coded labeling intervention (red = unhealthy, yellow = less healthy, green = healthy). Phase 2 increased the visibility and convenience of some green items. Duration: 2 years.	n/a	n/a	n/a	n/a	-Proportion of sales of red items decreased by 4% at F/U (p < 0.001) for both phases-Green sales increased by 5% at F/U (p < 0.001).-Red beverage sales decreased by 9% at F/U (p < 0.001).-Green beverage sales increased by 8% at F/U (p < 0.001).	Limited
(Thorndike et al., 2012), Location: Massachusetts, USA	Pre/Post. Hospital with 1 main cafeteria and 4 smaller on-site cafeterias. The intervention was carried out in the main cafeteria. The 2 on-site cafeterias were used as a comparison site with 1482 daily weekday transactions.	**✓***Targeting food choice at point of purchase*: Phase 1 was a 3-month color-coded labeling intervention (red = unhealthy, yellow = less healthy, green = healthy). Phase 2 added a 3-month choice architecture intervention that increased the visibility and convenience of some green items. Duration: 9 months.	n/a	n/a	n/a	n/a	-Sales of red items lowered by 9.2% in phase 1 and 4.9% in phase 2 (P < 0.001).-Sales of green items increased by 4.5% in phase 1 (P < 0.001).-Red beverage sales decreased by 16.5% during phase 1 (P < 0.001) and 11.4% in phase 2 (P < 0.001).- Green beverage sales increased by 9.6% in phase 1 (P < 0.001) and 4.0% in phase 2 (P < 0.001).-Bottled water sales increased by 25.8% in phase 2 (P < 0.001).	Limited
(Vyth et al., 2011), Location: Netherlands	Randomized trial. 25 (12: IG, 12: CG- 1 backup) worksite cafeterias with mainly sedentary employees. n = 1014.Mean age: 39.2 years.	**✓***Targeting food choice at point of purchase:* Choices logo was used to promote healthier eating for a 3-week period in IG cafeteria. Same sandwiches and soups were offered every day in addition to the Choices sandwich and soup. Choices logo was also placed on fresh fruit. Duration: 9 weeks.	CG cafeteria offered the same menu without the logo.	n/a	n/a	n/a	−1.159 units of fruits were sold per 50 lunching employees in the IG (95% CI: 0.454, 1.864; P = 0.001).–No intervention effects were found in the sales of sandwiches, soups, snacks and salads.	Limited
(Kottke et al., 2013), Location: Minneapolis, Minnesota, USA	Pre/Post. The corporate headquarters cafeteria of an integrated health system company with 2643 employees.	**✓***Targeting price:* Reduced price of salad bar purchases by 50%. The subsidy was publicized through an e-mail to all employees and by a large poster in the cafeteria. Duration: 1 month.	n/a	n/a	n/a	n/a	-Daily salad bar sales in March averaged 83% higher than sales averaged for other months (P = 0.008).−366% increase in salad bar sales by weight in March compared to other months representing a price elasticity of 7.32.	Limited
(Levy et al., 2012), Location: Massachusetts, USA	Pre/Post. Main cafeteria of hospital, open daily from 6:30am–8:00 pm. Participants had to be regular cafeteria patrons. On average weekdays, there were 6534 transactions totaling $31,404. Mean age: 41 years; Female: 71%.	**✓***Targeting food choice at point of purchase:* The first phase was a traffic light color-coded labeling system: healthy items (labeled green) and unhealthy items (labeled red). The second phase included “choice architecture” by physically rearranging certain cafeteria items, making green-labeled items more accessible.Duration: 9 months.	n/a	n/a	n/a	n/a	-Labeling decreased all employees red item purchases by − 11.2% (95% CI: −13.6%, −8.9%; P < 0.001) and increased green purchases by 6.6% (95% CI:5.2%, 7.9%; P < 0.001).	Limited
(Perlmutter et al., 1997), Location: Kansas, USA.	Pre/Post. Cafeteria of Kansas Farm Bureau and Affiliated Services serving 200 persons per day. All employees eating in the cafeteria were eligible to participate.	**✓***Targeting food quality*: 7 entrees from the cafeteria were modified to low total fat to < 30% of energy and sodium to < 1000 mg per serving and with nutrient information available. Modified standardized recipes and marketing of modified entrees was developed. **✓***Targeting client’s information, education or motivation*: Nutrient information was displayed on a large sign for all modified entrees being served that week. Duration: 7 months.	n/a	n/a	n/a	n/a	–No significant differences were observed in sales data.–No significant changes in overall acceptability were found for any entrée.	Limited
(Chen et al., 2017), Location: Taiwan	Time Series. National Health Research Institute, which had 1100–1200 employees. 220–330 lunches were sold on a typical day. Female: 55.2% (Survey 1), 56.4% (Survey 2).	**✓***Targeting food choice at point of purchase*: Dissemination of information on traffic light labelling. Phase 2: implementation of the traffic light labelling in the buffet. The labeling included red (unhealthy/stop), yellow (moderately healthy/wait) and green traffic light labels (healthy/go). Duration: 11 months.	n/a	n/a	n/a	n/a	-Proportion of customers who reported positive attitudes towards traffic light labelling increased by 12% (P < 0.01).-Proportion of buffet customers whose chose green light entrées increased by 23% (P < 0.001)-Red-light entrees choice decreased by 42% (P < 0.001).	Limited
(Levin, 1996), Location: Albuquerque, New Mexico	Non–-mized trial. Government employees at 2 urban worksites (1 IG and 1 CG site). n = 138 (IG); Mean age: 41 years; Female: 50%; Ethnicity: Hispanic: 60%, White: 30%.	**✓***Targeting food choice at point of purchase*: Poster on low-fat entrée selection and heart shaped labels were placed next to 3 targeted entrees (bean burritos, potato and chili burritos and a turkey, lettuce and tomato sandwich) on the menu board. Duration: 7 months.	No intervention in CG.	n/a	n/a	n/a	-The sales of the targeted low-fat entrees increased significantly at 6 weeks X^2^ = 50.24; p < 0.001) at the IG cafeteria.	Limited
(van Kleef et al., 2012), Location: Netherlands	Pre/Post. Dutch hospital staff canteen (field study only staff). About 500 people per weekday purchased items in the cafeteria. n = 92.	**✓***Targeting food choice at point of purchase:* Each week an alternative snack arrangement was on display i.e. 25% healthy at top shelves, 25% healthy at bottom shelves, 75% healthy at top shelves, and 75% healthy at bottom shelves. All products were sold at €0.85 except for fresh fruits (i.e., apples, oranges and bananas) which were sold at €0.50. All four conditions of assortment structures were displayed for one week. Duration: 4 weeks.	25% assortment structure, 25% shelf arrangement.	n/a	n/a	n/a	-For healthy snacks there was a significant main effect of assortment structure on sales, p = 0.01.- No significant effects of assortment structure, shelf arrangement or interaction on total sales.	Limited
(Sonnenberg et al., 2013), Location: Boston, Massachusetts, USA	Pre/Post. Main cafeteria at hospital. Opened seven days a week from 6:30 am to 8:00 pm, completing an average of 6534 transactions per weekday. n = 166 (BL), n = 223 (F/U); Female: 59%. Ethnicity: White 77%, Black 11%, Hispanic 6%, Asian 6%	**✓***Targeting food choice at point of purchase:* Traffic light labeling intervention in which all food and beverages served in the cafeteria were categorized by a negative or positive criterion. **✓✓✓***Targeting client’s information, education or motivation:* Dietitian support to answer questions about the labels and educate customers about the program. Pocket-sized pamphlets on labeling, calorie, fat, and saturated fat content of all items were supplied. Duration: 3 months.	n/a	n/a	n/a	n/a	-The proportion of respondents that identifiedhealth and nutrition as being an important factor in making their food or beverage choice increased by 20% (p = 0.004).-The proportion of respondents that reported looking at nutrition information increased by 18% (p < 0.0001).-Respondents who reported noticing the labels at the time of their purchase bought a higher proportion of green and lower proportion of red items compared to respondents who did not notice the labels (p < 0.001).	Limited
(Stites et al., 2015), Location: Pennsylvania, USA	Pre/Post. Hospital participants with a BMI of at least 25.0 kg/m^2^, ate 3 lunches a week at the cafeteria and had access to a computer at work. Participants diagnosed of unstable hypertension, dyslipidemia or coronary heart disease, whose medical therapy changed in the last 3 months, planned to terminate employment and was pregnant were excluded. n = 26. Mean age: 44.9 years;Female: 88.5%.	**✓***Targeting point of purchase*: The online pre-ordering system was designed to allow employees to order their lunches hours in advance of mealtime while viewing the nutrient content of the food choices. Daily and weekly specials rotated on a 4-week cyclic menu. **✓***Targeting price:* Participants were provided 20, $1.25 lunch vouchers for use in the 4-week full-intervention phase. **✓✓***Targeting client’s information, education or motivation*: Mindful eating training was provided to participants. Topics included the definition of mindful eating, education on different types of hunger, and tips on how to be more mindful.Duration: 4 weeks full intervention; and 4 weeks partial intervention.	Delayed treatment group CG.	n/a	n/a	n/a	-The treatment group purchased lunches with −144.6 fewer kilocalories (95% CI: −254.0, −351; p = 0.01).-The treatment group purchased meals had −8.9 fewer grams of fat per lunch meal (95% CI: −15.2, −2.6; p = 0.005) than the delayed-treatment group.	Limited
(Wolfenden et al., 2015),Location: New South Wales, Australia	Randomized trial.85 (IG = 43), (CG = 42) amateur community football clubs.(BL) n = 1394 (CG = 689, CG = 705). (F/U) n = 1134 (IG = 567, GC = 567).Mean age: 32.7 years (GG), 36.0 years (IG). Male: 87.0% (CG), 77.4% (IG).	**✓***Targeting food quality or quantity:* Provided a total of 6 FV (such as fresh fruit, salads or salad sandwiches) and non-sugar-sweetened drink products for sale at their club canteen. Substitution of high fat/energy products with low fat/energy products and introduce other ‘healthier’ products for sale. **✓***Targeting food choice at point of purchase:* Clubs were required to ensure at least 75% of non-alcoholic drinks in the canteen fridge were non-sugar-sweetened beverages and were positioned in the upper half of the fridge. Clubs were to ensure FV and non-sugar-sweetened drink products were displayed within view of consumers at all times.**✓***Targeting price*: Pricing strategies were encouraged to ensure that FV and non-sugar sweetened drink products were priced competitively compared to similar less healthy products.**✓***Targeting client’s information, education or motivation:* Promotional strategies to improve the physical environment included encouraging FV and non-sugar-sweetened drink purchase via meal deals, signage and posters to draw customer’s attention to such products. Duration: 27 months.	No intervention in CG. CG received printed material on topics unrelated to trial outcomes.	n/a	n/a	n/a	-Post-intervention, clubs receiving the intervention reported a significant increase in the availability of FV products (OR = 5.13; 95% CI: 1.70, 15.38; p = 0.006) compared to CG.-The proportion of intervention clubs offering meal deals and reduced pricing to promote FV products significantly increased following the intervention (OR = 34.48; 95% CI: 4.18, 250.00; p < 0.001) compared with CG.-The proportion of intervention club members reporting purchasing FV products increased significantly relative to members of CG clubs (OR = 2.58, 95% CI:1.08, 6.18; p = 0.03).-The purchase of non-sugar-sweetened drinks increased significantly amongst members of intervention clubs compared to members of CG (OR = 1.56; 95% CI: 1.09, 2.25; p = 0.01).	Limited
(Jeffery et al., 1994),Location: Minnesota, USA	Pre/Post.University office building with 700 employees isolated from alternative sources of prepared food. The cafeteria operated on weekdays and served fruit, cookies, sweet rolls, drinks, bread and snacks; and lunch items. Mean age: 38.7 years;Female: 62%.	**✓***Targeting food quality or quantity*: Doubling the number of fruit choices (6), increasing salad ingredient selections by 3. *Targeting price:* Reducing the price of fruit and salad by 50%. **✓✓✓***Targeting client’s information, education or motivation:* Advertisements of intervention posted in cafeteria and through employees’ mailbox. Duration: 9 weeks.	n/a	n/a	n/a	n/a	-Fruit and salad bar purchases increased markedly, p = 0.0001.	Limited
(Mazza et al., 2018) Location: Cincinnati, USA	Time series. Hospital medical center. Cafeteria handled 1200 transactions, representing $3,900 in daily revenue.	**✓***Targeting food quality or quantity:* Oppositional pairing of less healthy food with a healthy alternative. **✓***Targeting food choice at point of purchase:* Traffic light labeling, emoticons and health messages, social norm messages and color grouping. **✓***Targeting price*: Duration: 21 months.	CG: first 3 phases of the study including two price interventions and one labeling intervention	n/a	n/a	n/a	−2.9% point increase in healthy beverage sales with traffic light labeling (Phase2)(*p* < 0.0001) compared to Phase 1 (soda price increase. -Healthy beverage sales reduced: 2% (*p* < 0.0001) for color grouping (Phase 14), 1.7% (*p* < 0.01) for social norms group (Phase 10) and 6.9% (*p* = 0.01) for oppositional pairing (Phase 12). −5.4% increase in healthy chips sales (Phase 2) (p = 0.001) with traffic light labelling. −5.9% decrease of healthy chip sales (*p* = 0.003) with water price decrease and soda price increase (Phase 3) when added to the traffic light labeling.	Limited
(Viera et al., 2019) Location: North Carolina, USA	Pre/Post. 3 worksites with 371 participants. Participants needed to be a BCBSNC employee or contractor and eat lunch or be willing to eat lunch in the BCBSNC cafeteria at least 3 times per 5-day work week. Mean age: 42.2 years; Female: 78.4%. Ethnicity: 46% Black, 44 White, 9.7% Asian, 4.9% Hispanic	**✓***Targeting food choice at point of purchase:* One cafeteria received Physical Activity Calorie Expenditure (PACE)labels which showed the calories in the food as well as an image of someone walking and the estimated number of miles needed to “burn off” the calories. The two other cafeterias received calorie-only labels. Duration: 2 years.	Calorie-only labels.	n/a	n/a	n/a	Participants exposed to PACE labels purchased 40.4 fewer calories (p = 0.002), and participants exposed to calorie-only labels purchased 38.2 fewer calories (p = 0.0002).	Fair
(Thorndike et al., 2019) Location: Massachusetts, USA	Time series Massachusetts General Hospital with 5695 employees. Mean age: 40 years; Female: 71.2%. Ethnicity: Black 10%, Hispanic 7.2%, Asian 10.0%, White 72.8%.	**✓***Targeting food choice at point of purchase:* Traffic light food labels and choice architecture (product placement) changes. Duration: 2 years.	No comparator.	n/a	n/a	n/a	- Decrease of 19 kcal per transaction (95% CI: –23, −15 kcal) at 1 year from B/L and 35 kcal per transaction (95% CI: −39 ,-31; P < 0.001) at 2 years. -Red-labeled items decreased by 42 kcal per transaction at 2 years (95% CI: −45, −39 (–23%; *P* < 0.001). -Green-labeled items increased by 6 kcal per transaction (95% CI: 3, 9) (4%; *P* < 0.001)	Fair
(Pechey et al., 2019) Location: England	Randomized trial. Mix of office-based and depot/manufacturing worksites with 350 or more employees that could provide at least weekly sales data on individual items and the energy content of items sold.Mean age: 39.1 years;Female:33%.	**✓***Targeting food quality or quantity*: Increasing the proportion of healthier (i.e., lower energy) cooked meals, snacks, cold drinks and sandwiches while decreasing the number of less health options. Healthier cooked meals (excluding breakfast) were defined as having under 300 kcal for a meal component typically served with an additional potato or rice side or under 500 kcal for a complete meal. Healthier sandwiches were defined as those under 350 kcal. Healthy snacks were defined as savory snacks under 120 kcal per pack, sweet snacks under 150 kcal per pack and cold drinks under 50 kcal per pack. Duration: 5 months.	All 6 sites received intervention at different periods: Usual product availability (no intervention in CG) in site 2 for period 1, in site 3 for period 1 and 2, in site 4 for period 1, 2 and 3, in site 5 for period 1, 2, 3 and 4 and in site 6 for period 1, 2, 3, 4 and 5. Each period = 2 weeks.	n/a	n/a	n/a	-A reduction of 6.9% total daily energy purchased from targeted food categories for all sites (95% CI: −11.7, −1.7; p = 0.044). -Energy foods purchased significantly reduced in 2 sites by 10.7% (95% CI: −18.1, −2.6; p = 0.046) and 18.4% (95% CI: −26.9, −8.8; p = 0.013).	Fair
(Hollands et al., 2018) Location: England	Randomized trial. Nine worksites only six was included in data analysis. Worksites had to have ≥ 350 employees and could provide at least weekly sales data on individual items and the energy (kcal) content of items sold.Mean age: 38.9 years;Female: 34.9%.	**✓***Targeting food quality or quantity:* Reduce at least 10% the portion sizes of foods available in cafeterias from targeted categories (main meals, sides, desserts, cakes). Duration: 3–13 weeks.	Intervention varied by site with 6 to 49% of products altered at sites.	n/a	n/a	n/a	–No significant change when data from all 6 sites were pooled for daily energy purchased: −8.9% (95% CI: −16.7, −0.4; p = 0.081). -Reductions in energy purchased at sites ranged from −15.6 to −0.3%.	Fair
(Vasiljevic et al., 2018) Location: England	Randomized trial. 6 worksites with than 350 employees and had to provide weekly data on sales of individual items and their energy content. Mean age:25–34 years; Female: 46%.	**✓***Targeting food choice at point of purchase:* Labelling all cafeteria products for which such information was available with their calorie content (e.g., “250 Calories”) displayed in the same font style and size as for price. Duration: 17 weeks.	All 6 sites received intervention at different periods: No intervention in CG in site 2 for period 1, in site 3 for period 1 and 2, in site 4 for period 1, 2 and 3, in site 5 for period 1, 2, 3 and 4 and in site 6 for period 1, 2, 3, 4 and 5. Each period = 2 weeks.	n/a	n/a	n/a	-Null effect of overall intervention: −0.4% (95% CI: −3.8 , 2.9; p = 0.803). -Significant effect of intervention at 1 site: 6.6% reduction (95% CI: −12.9, − 0.3; p = 0.044) in energy purchased with calorie labelling.	Good
(Vasiljevic et al., 2019) Location: England	Randomized trial. 3 worksite cafeterias. Worksites had to have ≥ 300 employees and could provide at least weekly sales data on individual items and the energy (kcal) Female: 54%.	**✓***Targeting food choice at point of purchase:* Calorie content was prominently displayed in bold capitalized Verdana typeface with a minimum font size of 14 e.g., 120 calories. Duration: 6 weeks.	All three sites received intervention at different periods: No intervention in CG in site 2 for period 1 and 2 and for period 1, 2, 3 and 4 in site 3.	n/a	n/a	n/a	−87% of responding patrons wanted calorie labelling to remain in place. -Null effect on daily energy purchased: −0.6% (95% CI: −2.5, 1.2; p = 0.487).	Good

Effect	FV servings/day	Grams/day
Mild	<1	<80
Moderate	1–3	80–240
High	>3	>240

FV fruit and vegetable; BL Baseline; IG intervention group; CG control group; F/U follow up; n/a not assessed; WC waist circumference; SBP systolic blood pressure; Diastolic blood pressure; METs metabolic syndrome; BMI body mass index; EAP employment advisory board

#### 3.3.1 Changes in fruit and vegetable intake

There is evidence that workplace cafeteria and other supporting multicomponent interventions resulted in a higher intake of fruit and vegetables at the workplace. While five studies used single component interventions, 13 studies used multicomponent interventions in which 13 studies featured cafeteria-based interventions and five studies used both cafeteria and non-cafeteria interventions.

Using fruit and vegetable cut-offs to gauge effect; <1 serving/day < 80 g/day as mild, 1–3 serving/day 80–240 g/day as moderate and > 3 servings/day > 240 g/day as high, 16 out of the 18 studies showed a significant increase in fruit and vegetable intake (Bandoni et al., 2011, Beresford et al., 2000, Beresford et al., 2001, Buller et al., 1999, Cook et al., 2001, Emmons et al., 1999, Franco et al., 2013, Inoue et al., 2014, Kushida and Murayama, 2014, Lassen et al., 2014, Lassen et al., 2012, Lassen et al., 2011, Leighton et al., 2009, Thorsen et al., 2010, Uglem et al., 2013). Among these 16 studies, eight studies reported a moderate increase of 1–3 servings per day in fruit and vegetable consumption (Buller et al., 1999, Inoue et al., 2014, Lassen et al., 2012, Lassen et al., 2011, Leighton et al., 2009, Thorsen et al., 2010, Uglem et al., 2013). Four studies used a single component intervention, in which two studies used the intervention targeting food quality or quantity; the first offered a Japanese-style healthy lunch menu (Inoue et al., 2014) and the second increased the supply of fruit and vegetables (Leighton et al., 2009). The third study used the intervention targeting improved supply by training cafeteria staff (Thorsen et al., 2010), and remaining study used the intervention targeting client’s information, education or motivation through formal health communications methods (Buller et al., 1999).

Four studies used multicomponent interventions. One study featured the interventions targeting food quality and quantity, targeting price and targeting client’s information, education or motivation by offering healthy canteen choices, a free fruit program and information resources respectively (Lassen et al., 2011), one study featured the interventions targeting food quality and targeting price by offering free healthy takeaway meals (Lassen et al., 2012), and one study featured the intervention targeting food quality and targeting client’s information, education or motivation by offering a salad bar, increasing vegetable dishes and providing information about the health benefits of a diet rich in fruit and vegetables through posters (Uglem et al., 2013) and one study featured the interventions targeting food quality and quantity, targeting food choice at point of purchase, targeting price and targeting client’s information, education or motivation by introducing low energy dense foods and education thereof (Lowe et al., 2010).

Eight studies reported less than 1 serving per day increase in fruit and vegetable intake (Bandoni et al., 2011, Beresford et al., 2000, Beresford et al., 2001, Cook et al., 2001, Emmons et al., 1999, Franco et al., 2013, Kushida and Murayama, 2014, Lassen et al., 2014, Lassen et al., 2011) of which all featured multicomponent interventions except one study that used the intervention targeting client’s information, education or motivation through the placement of fruit and vegetable informational table tents at the cafeteria (Kushida and Murayama, 2014). Of the multicomponent intervention studies, five studies aimed to increase awareness on fruit and vegetable intake using posters or nutrition displays (Bandoni et al., 2011, Cook et al., 2001, Franco et al., 2013, Kushida and Murayama, 2014, Lassen et al., 2014), three studies offered cooking demonstrations and food tastings (Beresford et al., 2000, Beresford et al., 2001, Franco et al., 2013); four studies promoted or added healthy foods to their canteens menus (Beresford et al., 2000, Beresford et al., 2001, Cook et al., 2001, Lassen et al., 2014); two improved policy through written manuals (Bandoni et al., 2011, Emmons et al., 1999), two formed an employee advisory board (Beresford et al., 2000, Beresford et al., 2001); one provided free fruits (Franco et al., 2013); and two trained canteen staff on healthy eating and cooking (Bandoni et al., 2011, Franco et al., 2013).

#### 3.3.2 Changes in health risk indicators

While two studies used single component interventions, 14 studies used multicomponent interventions to affect changes in health risk indicators. Of these interventions, three studies used cafeteria-based interventions, while 13 studies used a combination of cafeteria and non-cafeteria interventions. In general, at least half of the studies had the expected benefits on health outcomes.

*Blood pressure*: Seven studies reported the effect on blood pressure. Out of these, four studies reported a significant reduction in systolic and diastolic blood pressure (Cook et al., 2001, Goetzel et al., 2010, Inoue et al., 2014, Leighton et al., 2009); two showed no significant difference (Ferdowsian et al., 2010, Geaney et al., 2016); whereas one showed a significant increase in blood pressure (Engbers et al., 2007). Using a single component intervention targeting food quality, a large reduction in blood pressure was observed after one year of the Mediterranean diet; SBP decreased by 13 mmHg and DBP decreased by 15 mmHg (Leighton et al., 2009) and in another study SBP decreased by 5.6 mmHg and DBP decreased by 7.6 mmHg through a Japanese style healthy lunch (Inoue et al., 2014). Two studies used multicomponent interventions with a significant reduction in systolic and diastolic blood pressure. Goetzel used the interventions targeting food quality, targeting food choice at point of purchase and targeting client’s information, education or motivation by changing the menu to promote healthy eating with point of choice prompts supplemented with staff counselling (Goetzel et al., 2010); and Cook used the interventions targeting food quality, targeting food choice at point of purchase and targeting client’s information, education or motivation by including low-fat meal options, offered water as a beverage, introduced point of choice messages promoting fruit and vegetables and installed nutrition displays in the cafeteria (Cook et al., 2001).

*Body Mass Index:* Out of ten studies that reported BMI (Brehm et al., 2011, Cook et al., 2001, Ferdowsian et al., 2010, Fernandez et al., 2015, Geaney et al., 2016, Goetzel et al., 2010, Iriyama and Murayama, 2014, LaCaille et al., 2016, Mishra et al., 2013b, Thorsteinsson et al., 1994), five studies showed a significant reduction in BMI (Ferdowsian et al., 2010, Fernandez et al., 2015, Geaney et al., 2016, Iriyama and Murayama, 2014, Mishra et al., 2013b). Three multicomponent intervention studies showed a significant reduction of greater than 1 kg/m^2^ (Ferdowsian et al., 2010, Geaney et al., 2016, Mishra et al., 2013b). Using the intervention targeting food quality, targeting price, targeting food choice at point of purchase and targeting client’s information, education or motivation Geaney reduced saturated fat, sugar and salt in meals, increased fruit and vegetables, discounted fruits, strategically positioned healthy alternatives and had monthly group nutrition presentations (Geaney et al., 2016), Mishra using the intervention targeting food quality and targeting client’s information, education or motivation implemented a low-fat plant-based diet in addition to weekly nutrition classes in the cafeteria (Mishra et al., 2013b) and Ferdowsian using the intervention targeting food quality and targeting client’s information, education or motivation offered low-fat vegan options and group presentations (Ferdowsian et al., 2010). Two multicomponent intervention studies showed a small significant reduction (less than 1 kg/m^2^) (Fernandez et al., 2015, Iriyama and Murayama, 2014). Fernandez included reduced sodium and calorie meals, fruit and vegetable subsidies, chef training workshop and brochures on nutrition (Fernandez et al., 2015). Iriyama included healthy meals in the menu and offered nutrition counselling (Iriyama and Murayama, 2014).

*Weight:* Nine studies reported change in weight (Cook et al., 2001, Ferdowsian et al., 2010, Fernandez et al., 2015, Goetzel et al., 2010, Iriyama and Murayama, 2014, LaCaille et al., 2016, Levin et al., 2010, Linde et al., 2012, Mishra et al., 2013b). Four multicomponent intervention studies reported a significant reduction on weight reduction (Ferdowsian et al., 2010, Iriyama and Murayama, 2014, Levin et al., 2010, Mishra et al., 2013b). A common intervention among these studies was the intervention targeting food quality or quantity; one study offered healthy food options to employees including 120 g of vegetables with restricted fat (Iriyama and Murayama, 2014); and three studies offered low-fat vegan meals (Brehm et al., 2011, Ferdowsian et al., 2010, Mishra et al., 2013b).

*Waist circumference:* Waist circumference was reported in six studies (Cook et al., 2001, Ferdowsian et al., 2010, Geaney et al., 2016, LaCaille et al., 2016, Leighton et al., 2009, Levin et al., 2010); three of them showed a significant reduction (Ferdowsian et al., 2010, Leighton et al., 2009, Levin et al., 2010). A common intervention among these studies was the intervention targeting food quality or quantity were healthy meals were offered. Two of the studies also featured the intervention targeting client’s information, education by raising awareness on healthy eating among workers.

*Lipids:* Out of seven studies that reported on change in HDL and LDL (Brehm et al., 2011, Engbers et al., 2007, Ferdowsian et al., 2010, Inoue et al., 2014, Leighton et al., 2009, Mishra et al., 2013b, Thorsteinsson et al., 1994) only three showed a small improvement in HDL levels (Engbers et al., 2007, Leighton et al., 2009, Thorsteinsson et al., 1994) and three showed a decrease in HDL (Brehm et al., 2011, Ferdowsian et al., 2010, Mishra et al., 2013b). LDL level decreased in three studies by less than 10 mg/dL (Brehm et al., 2011, Ferdowsian et al., 2010, Mishra et al., 2013b) and in one single component intervention study targeting food quality/quantity by 11 mg/dL by offering a Japanese style lunch (Inoue et al., 2014). Six studies reported on changes in total cholesterol (Brehm et al., 2011, Engbers et al., 2007, Goetzel et al., 2010, Inoue et al., 2014, Mishra et al., 2013b, Thorsteinsson et al., 1994); of which all reported a significant decrease in total cholesterol (Brehm et al., 2011, Engbers et al., 2007, Goetzel et al., 2010, Inoue et al., 2014, Mishra et al., 2013b, Thorsteinsson et al., 1994). Triglycerides were reported in two studies (Brehm et al., 2011, Leighton et al., 2009); one multicomponent intervention study showed a significant decrease in triglyceride levels (Brehm et al., 2011) by adding healthy entrees, increasing fruit and vegetable variety and replacing full fat with reduced fat items with the support of the employee advisory committee (Brehm et al., 2011).

*Glycated haemoglobin*: One multicomponent intervention study reported change in HbA1c (%) with a 0.7% reduction through a low-plant based diet in combination with weekly classes (Mishra et al., 2013b) whereas another multicomponent intervention study showed a significant reduction in fasting blood glucose through the improvement of the nutritional value of foods served in the cafeteria (Brehm et al., 2011).

*Metabolic syndrome:* One multicomponent study targeting improved supply and physical activity reported a decrease in the prevalence of metabolic syndrome by 9% among participants after implementing a cooking course to chefs of the cafeteria (Hjarnoe and Leppin, 2013).

#### 3.3.3 Changes in other dietary intake

While three studies used single component interventions, 17 studies used multicomponent interventions to affect changes in dietary intake. Among these interventions, 13 studies used cafeteria-based interventions and seven studies used a combination of cafeteria and non-cafeteria interventions. There is evidence that changing the food environment resulted in improved dietary intake at the workplace.

*Total fat*: Four studies (Bandoni et al., 2011, Berkowitz et al., 2016, Geaney et al., 2010, Levin et al., 2010, Vanderlee and Hammond, 2014) reported a significant decrease in total fat intake out of which two studies had a greater than 15 g reduction in total fat intake (Geaney et al., 2010, Levin et al., 2010). Both studies used multicomponent interventions targeting food quality or quality and targeting client’s information, education or motivation, one study included low fat vegan menu options and provided cooking demonstrations (Levin et al., 2010); another restricted food high in fat, limited cooking methods with oil and provided nutrition information (Geaney et al., 2010).

*Saturated fat:* Five studies reported a decrease in saturated fat intake (Berkowitz et al., 2016, Brehm et al., 2011, Geaney et al., 2010, Geaney et al., 2016, Levin et al., 2010, Vanderlee and Hammond, 2014). Common to these five studies was the intervention targeting food quantity or quality, one study introduced low fat vegan menu options (Levin et al., 2010), two reduced foods high in fat (Geaney et al., 2010, Geaney et al., 2016), two conducted taste tests to modify healthy meals (Geaney et al., 2016) and one introduced reduced size entrees (Berkowitz et al., 2016).

*Fiber:* Eight studies reported a significant increase in fiber intake (Bandoni et al., 2011, Emmons et al., 1999, Ferdowsian et al., 2010, Inoue et al., 2014, Lassen et al., 2011, Levin et al., 2010, Mishra et al., 2013a, Mishra et al., 2013b). Four studies had a common intervention targeting food quantity and quality to increase fruit and/or vegetables consumption through a low-fat vegan menu (Ferdowsian et al., 2010, Levin et al., 2010, Mishra et al., 2013a, Mishra et al., 2013b), one study labelled healthy food (Emmons et al., 1999), one study conducted culinary workshops for canteen operators (Bandoni et al., 2011), one study offered a Japanese style lunch with increased vegetables (Inoue et al., 2014) and one study had a free fruit program (Lassen et al., 2011).

*Total energy intake*: Five studies reported on energy intake, found significant reductions (Berkowitz et al., 2016, Inoue et al., 2014, Lassen et al., 2014, Lassen et al., 2012, Levin et al., 2010, Vanderlee and Hammond, 2014).

*Sugar products:* One study provided cafeteria staff with a healthy cooking course, resulting in reduced intake of sugar products (Hjarnoe and Leppin, 2013).

*Whole grains:* One study resulted in an increased consumption of whole grain bread by improving the whole grain content of bread by 50–100% and fiber content of 4–7 g/100 g at meals (Uglem et al., 2013).

#### 3.3.4 Changes in food sales

From the 24 studies that assessed changes in sales of healthy food, 17 studies used single component interventions while seven studies used multicomponent interventions. Among these interventions, 20 studies used cafeteria-based interventions, while four studies used a combination of cafeteria and non-cafeteria interventions. Eighteen studies using interventions targeting food choice at point of purchase increased sales of healthy foods (Chen et al., 2017, Jeffery et al., 1994, Kottke et al., 2013, Levin, 1996, Levy et al., 2012, Mazza et al., 2018, Sonnenberg et al., 2013, Steenhuis et al., 2004, Steenhuis et al., 2004, Stites et al., 2015, Thorndike et al., 2019, Thorndike et al., 2014, Thorndike et al., 2012, van Kleef et al., 2012, Vasiljevic et al., 2018, Vasiljevic et al., 2019, Viera et al., 2019, Vyth et al., 2011, Wolfenden et al., 2015). Thirteen of the 24 studies reported significant increase in sales of healthy food and beverages. Seven studies used traffic light labelling (Chen et al., 2017, Levy et al., 2012, Mazza et al., 2018, Sonnenberg et al., 2013, Thorndike et al., 2019, Thorndike et al., 2014, Thorndike et al., 2012), and two studies used healthy symbol labels (Levin, 1996, Vyth et al., 2011) of which one significantly increased fruit sales but had no impact on the sale of sandwiches, soups and salads using the healthy symbol (Vyth et al., 2011). Four studies with significantly increased sales used interventions targeting price. One study increased sales of healthy food through the reduction of salad bar prices by 50% (Kottke et al., 2013), one study offered meal vouchers (Stites et al., 2015), one study offered competitive pricing of healthy drinks (Wolfenden et al., 2015) and one study reduced the price of fruits and salad by 50% (Jeffery et al., 1994).

#### 3.3.5 Behavioral change

From the 31 multicomponent intervention studies, twenty four studies used all three essential conditions; (Beresford et al., 2000, Beresford et al., 2001, Cook et al., 2001, Engbers et al., 2006, Engbers et al., 2007, Ferdowsian et al., 2010, Fernandez et al., 2015, Franco et al., 2013, Geaney et al., 2010, Geaney et al., 2016, Goetzel et al., 2010, Jeffery et al., 1994, LaCaille et al., 2016, Lassen et al., 2011, Levin et al., 2010, Linde et al., 2012, Lowe et al., 2010, Perlmutter et al., 1997, Sonnenberg et al., 2013, Steenhuis et al., 2004, Steenhuis et al., 2004, Stites et al., 2015, Thorsteinsson et al., 1994, Uglem et al., 2013);Mishra et al., 2013b) of which four studies produced a null result (Engbers et al., 2006, LaCaille et al., 2016, Linde et al., 2012, Perlmutter et al., 1997). Among the 24 single component intervention studies one or two of the essential conditions were used and two studies did not report a positive result (Vasiljevic et al., 2018, Vasiljevic et al., 2019).

### 3.4 Quality assessment

The assessment of the quality of included studies was impeded by incomplete reporting, and consequently, an unclear risk of bias judgement was reached for some domains. Six out of 55 studies were graded as good quality studies, 14 studies as fair quality and 35 studies as limited quality.

## 4 Discussion

The results of this systematic review demonstrate that cafeteria interventions and supporting non-cafeteria interventions at worksites promote healthy eating and influence health-related behaviors among adults. There is evidence that workplace cafeteria and other supporting multicomponent interventions resulted in a higher intake of fruit and vegetables, improved dietary intake, improved health outcomes and improved healthy food sales at the workplace. Several studies used multicomponent interventions, and the most featured interventions included interventions targeting food quality or quantity, targeting client’s information, education or motivation and targeting food choice at point of purchase.

In this review, sixteen out of 18 studies demonstrated a mild to moderate effect in the increase of fruit and vegetable intake. Of those that were effective, most studies used interventions targeting food quality or quantity and client’s information, education or motivation. Mechanisms for increasing fruit and vegetable intake included expanding fruit and vegetable availability and the provision of informational material on healthy eating. Likewise, two other reviews reported a positive impact on fruit and vegetable consumption through multiple component intervention strategies (Hendren and Logomarsino, 2017, Ni Mhurchu et al., 2010). In this review, at least half of the workplace cafeteria and other supporting multicomponent interventions had the expected benefits on health outcomes. Fourteen out of 16 studies that evaluated change in risk factors demonstrated a positive effect on either blood pressure, BMI, weight, WC, lipid, glycated haemoglobin or metabolic syndrome. Most studies used a combination of interventions, targeting food quality or quantity and targeting client’s information, education or motivation. Mechanisms to affect changes in health risk indicators included introducing healthier cafeteria foods with reduced fat, vegan options, and lifestyle education. In this review, there is evidence that workplace cafeteria and other supporting multicomponent interventions result in improved dietary intake at the workplace. Eighteen out of 20 studies that evaluated changes in dietary intake, reported a significant effect on total fat, saturated fat, fiber and total energy intake. Most studies used a combination of interventions targeting client’s information, education or motivation and food quality or quantity. Mechanisms for reducing fat with positive changes in dietary intake included low-fat vegan menu options, restriction of food high in fat, limiting cooking methods requiring oil and offering low-fat meal options. In this review, 13 out of 24 studies reported a significant increase in the sale of healthy food and beverages using environmental level changes; labelling and pricing. Most studies used cafeteria-based interventions targeting food choice at point of purchase. The review by Al-Khudairy, on choice architecture intervention to improve dietary behavior found that there was no strong evidence for the effect of pricing and on labelling alone on behavioral change (Al-Khudairy et al., 2019). However, interventions including the availability and proximity element were generally reported to be successful in changing behaviour (Al-Khudairy et al., 2019).

Given that most studies reported positive results using either all or one to two behaviour conditions, it is important that when selecting interventions, it has to be mapped to the behaviour target for intervention success. In general, the included studies were of fair to limited quality. Six studies were graded as good quality. The strength of studies could have been comprised due to the inherent limitations of a worksite setting and adherence to interventions. Moreover, our evaluation of the quality of studies was impeded by incomplete reporting.

This review has several strengths and limitations. We did a comprehensive search, covering more than 20 years of research including all types of worksites which improves the generalizability of the findings, however it is possible the search did not identify all studies published. Furthermore, the review study search was restricted to studies published in English and excluded unpublished studies. We assessed the quality of the studies using a standard quality assessment tool, with the built-in flexibility of assessing the quality of different study designs. The primary limitation of this review was the heterogeneity of the study designs, outcomes and outcome measures among studies which limited data pooling to perform a meta-analysis, hence limiting the direct comparison of studies to quantify the results to assess the effectiveness of specific interventions. Reporting the results by intervention type should be considered for future research to highlight the exact effect by intervention type to promote healthy eating and reductions in health risks. Several trails produced multiple papers; hence it is suggested that different papers that belong to same trail be reported together.

## 5 Conclusion

The review has the potential to inform future workplace health interventions in tackling workplace obesogenic environments and promoting positive dietary behavior changes. Understanding the components and processes included in such interventions has implications to inform employers and implementers about intervention options, components, format, duration and opportunities that exist to improve the health of a workforce. Future research should standardize the intervention assessment tool, outcome measures as well as evaluate the sustainability of the interventions in terms of cost and acceptability of interventions by employees. This will improve the quality of evidence available and allow for thorough assessment to identify the most effective interventions and implementation strategies. Multicomponent interventions, specifically interventions targeting food quality or quantity, interventions targeting client’s information, education or motivation and interventions targeting food choice at point of purchase have the potential to produce positive health related behaviors at worksites.

## Funding sources

This research is funded by: NIH:115773932 and NIH:5DP1ES02545903.

## Declaration of Competing Interest

The authors declare that they have no known competing financial interests or personal relationships that could have appeared to influence the work reported in this paper.
