# Supplementary material for: Workplace cafeteria and other multicomponent interventions to promote healthy eating among adults: A systematic review
Source: Prev Med Rep. 2021 Feb 23;22:101333. doi: 10.1016/j.pmedr.2021.101333 (PMC7937753; doi:10.1016/j.pmedr.2021.101333)
Supplement: Supplementary data 1 [file mmc1.docx]

| **Section/topic** | **#** | **Checklist item** | **Reported on page #** | **Text** |
| --- | --- | --- | --- | --- |
| **TITLE** | | | | |
| Title | 1 | Identify the report as a systematic review, meta-analysis, or both. | 1 | Workplace cafeteria and other multicomponent interventions to promote healthy eating among adults: A systematic review |
| **ABSTRACT** | | | | |
| Structured summary | 2 | Provide a structured summary including, as applicable: background; objectives; data sources; study eligibility criteria, participants, and interventions; study appraisal and synthesis methods; results; limitations; conclusions and implications of key findings; systematic review registration number. | 2 | Please see the ABSTRACT |
| **INTRODUCTION** | | | | |
| Rationale | 3 | Describe the rationale for the review in the context of what is already known. | 3 | Several systematic reviews have been conducted that evaluate the effectiveness of worksite health promotion trials. However, results from one review found that there are few studies that focused on the impact of food environmental modifications on dietary intakes and that the few studies containing an environmental component obtained inconclusive results. In addition, it proves challenging to filter out successful intervention components in changing dietary behaviour. |
| Objectives | 4 | Provide an explicit statement of questions being addressed with reference to participants, interventions, comparisons, outcomes, and study design (PICOS). | 4 | Workplace (P) cafeteria interventions and other supporting multicomponent interventions (I) – Cafeteria: Targeting food quality and quantity, targeting food choice at point of purchase, targeting price, targeting organizational polices, targeting improved supplies, targeting clients information, education or motivation, non-cafeteria (supporting interventions) or both  (C) Control group (O) are effective in promoting healthy eating and reducing health risks among adults?  Hence, this systematic literature review aims to identify and assess the effectiveness of workplace cafeteria interventions on fruit and vegetable intake, other aspects of dietary intake, health outcomes and changes in food sales. |
| **METHODS** | | | | |
| Protocol and registration | 5 | Indicate if a review protocol exists, if and where it can be accessed (e.g., Web address), and, if available, provide registration information including registration number. | 4 | No |
| Eligibility criteria | 6 | Specify study characteristics (e.g., PICOS, length of follow-up) and report characteristics (e.g., years considered, language, publication status) used as criteria for eligibility, giving rationale. | 4 | Please see search strategy and procedures. |
| Information sources | 7 | Describe all information sources (e.g., databases with dates of coverage, contact with study authors to identify additional studies) in the search and date last searched. | 4 | We searched multiple databases including EMBASE (general medicine), CINAHL (nursing & allied health), EconLit, Ovid, Cochrane, Web of Science and PubMed.  The date of search for each database is provided in S2 File. |
| Search | 8 | Present full electronic search strategy for at least one database, including any limits used, such that it could be repeated. | 4 | MeSH search terms included: (1) Setting-based: cafeteria, canteen, school, workplace, worksite, campus, industry; (2) Intervention-based: nutrition, diet, dietary intervention, health promotion, primary prevention, health behaviour, health education, food, program evaluation. The complete list of the search terms is provided in the S2 File. |
| Study selection | 9 | State the process for selecting studies (i.e., screening, eligibility, included in systematic review, and, if applicable, included in the meta-analysis). | 4 | Please see search strategy and procedures |
| Data collection process | 10 | Describe method of data extraction from reports (e.g., piloted forms, independently, in duplicate) and any processes for obtaining and confirming data from investigators. | 4-5 | Two researchers (AN, CJ) screened the titles and abstracts, and full paper if necessary separately and independently using a screening verification checklist with five possible outcomes; (a) exclude - not relevant to review question, (b) exclude - fails to meet the inclusion criteria and in this case the reason for exclusion was noted, (c) exclude – duplicate, (d) include, and (e) unsure. Any disagreements and unsure studies regarding inclusion were resolved by discussion with the third researcher (AS) until consensus was reached.  A copy of the full text of papers were obtained for each of the included studies. The screening checklist was re-applied in assessing the content of the paper. Studies not meeting the review inclusion criteria were excluded however, studies meeting the inclusion criteria and belonging to same trial was included. Two reviewers (AN and CJ) independently extracted information from 56 studies each using the Data Abstraction Form published by the Guide to Community Preventive Services to classify and describe key characteristics of the intervention. A third reviewer (AS) double checked 20% of the extracted studies for accuracy of data extraction. Thereafter, extraction results were compared for agreement and differences regarding data extraction were resolved by discussion until consensus was reached by all reviewers. |
| Data items | 11 | List and define all variables for which data were sought (e.g., PICOS, funding sources) and any assumptions and simplifications made. | 5 | Data were extracted using the Data abstraction Form published by the Guide to Community Preventive Services |
| Risk of bias in individual studies | 12 | Describe methods used for assessing risk of bias of individual studies (including specification of whether this was done at the study or outcome level), and how this information is to be used in any data synthesis. | 5 | Quality of study execution included an evaluation of five categories of threats to validity; study population and intervention descriptions, sampling, exposure and outcome measurement, data analysis, interpretation of results and other biases, based on the Guide to Community Preventive Services guide which allows for the evaluation of different study designs with questions to evaluate a general concept. All studies that met the inclusion criteria were assessed by the two reviewers independently for their methodologic quality, following the predefined checklist of questions that assess potential threats to the validity of each study (S5 File). The reviewers scored the item as positive (+) if the item was met, negative (-) if the item was not met, and unclear (?) if insufficient information was provided. The total quality score was calculated by counting the number of items scored positively. Studies with none or one limitation was classified as good, studies with 2-4 limitations were classified as fair and studies with 5 or more limitations were classified as limited (Briss et al., 2000). Results were compared for agreement and differences regarding the quality score were resolved by discussion until consensus was reached by the reviewers. |
| Summary measures | 13 | State the principal summary measures (e.g., risk ratio, difference in means). | 7 | The primary outcome was the percent change in fruit and vegetable intake, dietary intake, health risk indicators and sales of food. |
| Synthesis of results | 14 | Describe the methods of handling data and combining results of studies, if done, including measures of consistency (e.g., I^2^) for each meta-analysis. | - | - |
| Risk of bias across studies | 15 | Specify any assessment of risk of bias that may affect the cumulative evidence (e.g., publication bias, selective reporting within studies). | 7, 8 | - |
| Additional analyses | 16 | Describe methods of additional analyses (e.g., sensitivity or subgroup analyses, meta-regression), if done, indicating which were pre-specified. | - | - |
| **RESULTS** | | | | |
| Study selection | 17 | Give numbers of studies screened, assessed for eligibility, and included in the review, with reasons for exclusions at each stage, ideally with a flow diagram. | Fig 1 | Fig 1 |
| Study characteristics | 18 | For each study, present characteristics for which data were extracted (e.g., study size, PICOS, follow-up period) and provide the citations. | Table1 and Table 2 | Please see Table 1 and Table 2 |
| Risk of bias within studies | 19 | Present data on risk of bias of each study and, if available, any outcome level assessment (see item 12). | S File 6 | Please see Supplementary File 6.  We assessed methodological quality using five categories; description, sampling, exposure and outcome measurement, data analysis, interpretation of results and other limitations (S6 File). The assessment of the quality of included studies were impeded by incomplete reporting and consequently an unclear risk of bias judgement was reached for some domains. Six out of 55 studies were graded as good quality studies, 14 studies were graded as fair quality and 35 studies were graded as limited quality. |
| Results of individual studies | 20 | For all outcomes considered (benefits or harms), present, for each study: (a) simple summary data for each intervention group (b) effect estimates and confidence intervals, ideally with a forest plot. | Table 2 and S File 5, | Please see Table 2 and S File 5 |
| Synthesis of results | 21 | Present results of each meta-analysis done, including confidence intervals and measures of consistency. | - | - |
| Risk of bias across studies | 22 | Present results of any assessment of risk of bias across studies (see Item 15). | 10 | We assessed methodological quality using five categories; description, sampling, exposure and outcome measurement, data analysis, interpretation of results and other limitations (Zaza et al., 2000) (Table S6 File). The assessment of the quality of included studies were impeded by incomplete reporting and consequently an unclear risk of bias judgement was reached for some domains. Six out of 55 studies were graded as good quality studies, 14 studies were graded as fair quality and 35 studies were graded as limited quality. |
| Additional analysis | 23 | Give results of additional analyses, if done (e.g., sensitivity or subgroup analyses, meta-regression [see Item 16]). | - | - |
| **DISCUSSION** | | | | |
| Summary of evidence | 24 | Summarize the main findings including the strength of evidence for each main outcome; consider their relevance to key groups (e.g., healthcare providers, users, and policy makers). | 5-10 | See pages 5-10 |
| Limitations | 25 | Discuss limitations at study and outcome level (e.g., risk of bias), and at review-level (e.g., incomplete retrieval of identified research, reporting bias). | 12 | This review has several strengths and limitations. We did a comprehensive search, covering more than 20 years of research and included all kinds of worksites; however it is possible the search did not identify all studies published, which have could have led to selection bias. Furthermore, the review study search was restricted to studies published in English. Unpublished studies are an important part of the knowledge base. We acknowledge the criterion to exclude unpublished studies as a limitation. The studies included in this review implemented interventions across multiple worksites, which improves the generalizability of the findings. We assessed the quality of the studies using a standard quality assessment tool, with the built-in flexibility of assessing the quality of different study designs. The primary limitation of this review was the heterogeneity of the study designs, outcomes and outcome measures among studies which limited data pooling to perform a meta-analysis, hence limiting the direct comparison of studies to quantify the results to assess the effectiveness of specific interventions. Most of the studies in this review did not randomly assign the intervention, which can introduce confounding bias. The non-random assignment and absence of blinding poses concerns regarding internal validity, however, within a real-life worksite setting, these are more practical considering problems of contamination due to employee contact. Some studies used convenience sampling, therefore, were not free from selection bias and may not completely represent the target population. There was heavy reliance on self-reported dietary data, which is subject to measurement error, recall bias, and under and over-reporting. Future worksite dietary interventions studies should include objectives measures of dietary behavior |
| Conclusions | 26 | Provide a general interpretation of the results in the context of other evidence, and implications for future research. | 12 | The review has the potential to inform future workplace health interventions in tackling workplace obesogenic environments and promoting positive dietary behavior changes through multiple component intervention strategies. Understanding the components and processes included in such interventions has implications to inform employers about intervention options, components, format, duration and opportunities that exist to improve the health of their workforce. Future research should standardize the intervention assessment tool, outcome measures as well as evaluate the sustainability of the interventions. This will improve the quality of evidence available and allow for thorough assessment to identify the most effective interventions and implementation strategies. More research is warranted on solution-oriented, real-life setting research that also takes into consideration the motivation and empowerment of the stakeholders to provide sustainable improvements in worksite food environments for healthy eating. A range of interventions strategies has been tested to improve food environments and health outcomes in worksite settings. Multicomponent food environment interventions, specifically interventions targeting food quality or quantity, interventions targeting client’s information, education or motivation and interventions targeting food choice at point of purchase have the potential to produce positive health related behaviors at worksites. |
| **FUNDING** | | | | |
| Funding | 27 | Describe sources of funding for the systematic review and other support (e.g., supply of data); role of funders for the systematic review. | 12 | This research is funded by: NIH:115773932 and NIH:5DP1ES02545903. |

*From:*  Moher D, Liberati A, Tetzlaff J, Altman DG, The PRISMA Group (2009). Preferred Reporting Items for Systematic Reviews and Meta-Analyses: The PRISMA Statement. PLoS Med 6(6): e1000097. doi:10.1371/journal.pmed1000097

For more information, visit: **www.prisma-statement.org**.
