# Supplementary material for: Workplace cafeteria and other multicomponent interventions to promote healthy eating among adults: A systematic review
Source: Prev Med Rep. 2021 Feb 23;22:101333. doi: 10.1016/j.pmedr.2021.101333 (PMC7937753; doi:10.1016/j.pmedr.2021.101333)
Supplement: Supplementary data 3 [file mmc3.docx]

**S3 File**

**Table S3.** Classification of intervention strategies.

| **Interventions** |
| --- |
| **Interventions targeting food quality/ quantity** |
| Remove/reduce trans fat |
| Reduce saturated fat |
| Reduce sugar |
| Reduce the amount of salt during cooking |
| Increase fruit and vegetable choices |
| Reduce portion sizes of foods |
| Offer smaller portion size with proportionate pricing |
| Ready to eat healthy meals |
| Reduce soda |
| Free clean cold water |
| Add salad bar |
| **Interventions targeting price** |
| Price discounts for whole fresh fruit |
| Provide free fruit and/or vegetables |
| Healthier foods such as fruits, fresh juice, whole grains for reduced price |
| Fixed price for all menus |
| Provision of meal vouchers for healthy meals |
| **Interventions targeting food choice at point of purchase** |
| Traffic Light Labelling |
| Strategic positioning of healthier alternatives to make healthy items more accessible such as keeping fruits at the eye level |
| Healthy option station |
| Use a nutrition logo or a specific symbol on healthy food to help people recognize which foods are healthy |
| Display the number of calories of a product translated into the number of minutes to perform a certain physical activity at work (e.g. 35 minutes’ walk for a can of soda) |
| **Interventions targeting improved supply** |
| Train managers to increase the availability of healthy food choices in his/her worksite cafeteria. |
| Culinary workshops for cafeteria workers, chefs and kitchen assistants on preparing healthy meals, using fruit and vegetables, presentation, arrangement |
| **Interventions targeting client’s information, education or motivation** |
| Healthy lunchtime clubs |
| Food workshops to discuss healthy foods and taste demonstrations of healthy food options |
| Informational material (e.g. nutrition quizzes, dinner mats, computer-based activities, leaflets) |
| Monthly news magazine with information on healthy food options |
| Educational materials distribution with messages encouraging fruit and vegetable consumption |
| A nutrition resource kiosk with trained personnel available to discuss about healthy diet and provide resources |
| Posters, napkins, a self-evaluation brochure |
| Cooking demonstrations and tasting healthy foods |
| Lifestyle education |
| **Interventions targeting organization policies** |
| Develop/ Modify organizational health policy on promotion of healthy eating |
| Develop a manual aimed at the cafeteria’s managers on worker’s food program, nutritional guidelines, importance of a balanced diet highlighting the key role of fruit and vegetables |
| An employee advisory board (EAB) to guide the planning and implementation of change to help make the cafeteria healthier |

**S4 File**

**Table S4.** Categories of questions that assess potential threats to the validity of each study.

| **Criteria** |
| --- |
| Descriptions |
| 1. Was the study population well described? |
| 1. Was the intervention well described (what, how, who, where)? |
| Sampling |
| 1. Was the sampling frame or universe of selection for the study purpose specified? |
| 1. Were the screening criteria for study eligibility specified? |
| 1. Was the population that served as the unit of analysis the entire eligible population? |
| Measurements |
| 1. Was there an attempt to measure exposure to intervention? |
| 1. Were exposure variables valid measures? |
| 1. Were exposure measures reliable? |
| 1. Were outcome and other independent variables valid measures? |
| 1. Were the outcome and other independent variables reliable? |
| Data analysis |
| 1. Did the authors conduct statistical testing? |
| 1. Did authors report on statistical test used? |
| 1. Did authors control for design effects in the statistical model? |
| 1. Did authors control for repeated measures in the analysis? |
| 1. Did authors account for different level of exposure? |
| Interpretation of results |
| 1. Did 80% participants complete the study? |
| 1. Were units of analysis comparable prior to the exposure of the intervention? |
| 1. Were appropriate methods for controlling confounding variables and limiting potential bias used? |

**S5 File**

**Table 3.** Summary of studies reporting intervention components with behavioral element and by outcome.

| **Study and study design** | **Intervention components** | | | | | | **Outcome** | | | |
| --- | --- | --- | --- | --- | --- | --- | --- | --- | --- | --- |
|  | **Food quality or quantity** | **Price** | **Food choice** | **Improved supply** | **Client’s information, education or motivation** | **Organizational policies** | **Changes in FV consumption** | **Changes in health risk indicators** | **Changes in dietary intake** | **Changes in food sales** |
| (Bandoni et al., 2011) RCT |  |  |  | Culinary workshops for cafeteria workers. (*Training*: Capability) | Educational materials distributed at cafeteria to encourage FV consumption and poster displays to summarize the main points of the previous intervention strategies.  (*Training*: Capability;  *Persuasion*: Motivation) | Production of a manual for cafeteria managers. (*Enablement:* Capability/ Opportunity) | ↑ FV | n/a | ↓Total fat, ↑Fiber | n/a |
| (Beresford et al., 2001) RCT | Provisions of more FV as part of the regular menus. (*Environmental restructuring:* Opportunity) |  |  |  | Worksite wide educational opportunities (taste tests, cooking demonstrations).  (*Training* and *Education:* Capability; Modelling: Motivation and Capability) | An employee advisory board was set up to support changes at worksites.  (*Enablement:* Capability/ Opportunity) | ↑FV | n/a | n/a | n/a |
| (Engbers et al., 2006) Non- randomized trial | Every 2 months for 1 day a week a healthy buffet was offered.) (*Environmental restructuring:* Opportunity) |  | Placement of informational sheets near food products with caloric value translated into the number of minutes to perform a certain activity. (*Persuasion*: Motivation) |  | An information stand was placed in the canteen with brochures and leaflets on healthy food, blood pressure and cholesterol.  *(Education:* Capability) |  | Null result | n/a | Null result | n/a |
| (Franco et al., 2013) Pre/Post |  | Workers were provided with a meal voucher. Fruit and desserts were sold at a fixed price. (*Coercion:* Opportunity/ Motivation) |  | The canteen operator and the nutritionist were made more aware of the importance of promoting FV. The nutritionist created menus and supervised the production of meals.  (*Training*: Capability) | A food tasting stand was set up showcasing FV dishes. Table displays were set up to promote FV consumption.  *(Education:* Capability; Persuasion: Motivation) |  | ↑FV | n/a | n/a | n/a |
| (Beresford et al., 2000) RCT | Structural changes in food availability to provide more FV as part of their regular menus.  (*Environmental restructuring:* Opportunity) |  |  |  | Regular message posting about 5-a-Day and worksite wide educational opportunities (taste tests and cooking demonstrations).  *(Education:* Capability; *Persuasion*: Motivation; Modelling: Motivation and Capability) | The formation of an employee advisory board at each work site.  *(Enablement:* Capability/ Opportunity) | ↑FV | n/a | n/a | n/a |
| (Thorsen et al., 2010) Pre/Post |  |  |  | An 8-hour training session for all canteen staff. Goal setting (average grams of total FV consumed per customer per meal) by canteen staff.  (*Training and Education*: Capability) |  |  | ↑FV | n/a | n/a | n/a |
| (Buller et al., 1999) RCT |  |  |  |  | Five a Day Education Program using standard formal communication channels (e.g. workplace mail, cafeteria promotions and speakers).  *(Education:* Capability;  *Persuasion*: Motivation) |  | ↑FV | n/a | n/a | n/a |
| (Kushida and Murayama, 2014) Non- randomized trial |  |  |  |  | At the IG sites, 12 types of informational table tents were placed once every 2 weeks on all tables in each cafeteria. Information about vegetable consumption was presented in stages.  *(Education:* Capability;  *Persuasion*: Motivation) |  | ↑Vegetable | n/a | n/a | n/a |
| (Uglem et al., 2013) RCT | A self-service salad bar consisting of a large variety of vegetables was introduced for the lunch meal. For dinner, vegetables were included in newly developed dishes, or vegetables were offered as side dishes. Bread with a wholegrain content of 50-100%, and a fiber content of 4 -7 g/100 g was offered at all meals. (*Environmental restructuring:* Opportunity) |  |  |  | Information about the health benefits of a diet rich in FV and whole grain cereals were given to the recruits in an information meeting and through posters, brochures and folders. 3 different posters, 5 of each, were present at the same time, being replaced with new versions every 6 weeks, containing information about main health effects of vegetables and whole grain bread. *(Education:* Capability; *Persuasion*: Motivation) |  | ↑Vegetable | n/a | ↑Whole grain consumption | n/a |
| (Leighton et al., 2009) Pre/Post | The food offer during the intervention period included a salad bar presented everyday with different mixed salads, plus 2 options for main dish and natural fruits as a dessert. To encourage salad consumption an olive oil-based salad dressing containing herbs and spice was continuously available. Mediterranean diet menu, a vegetarian dish was available plus an option of beef with rice for consumers not interested in adhering to the Mediterranean diet. An olive oil bottle was routinely available at the salad bar counter. (*Environmental restructuring:* Opportunity) |  |  |  |  |  | ↑FV | ↓WC, SBP, DBP, ↑HDL, NS blood glucose, triglyceride | ↑Mediterranean diet score | n/a |
| (Thorsteinsson et al., 1994) RCT | Menu ingredient changes included whole milk replaced with skimmed milk, a bread spread with less fat and fiber rich bread. Fat content of the lunch meals was decreased, and vegetables and salads were added to the menu. Lunches were calculated at about 1000 kcal, breakfast about 700-800 kcal, and the bread and biscuits in the coffee breaks contained fewer calories.  (*Environmental restructuring:* Opportunity;  *Restriction:* Opportunity) |  |  |  | Included consultations, written instructions, additional blood lipid measurements and meetings with the dietitian.  *(Education:* Capability; *Persuasion*: Motivation) |  | n/a | ↓Cholesterol, ↑HDL, NS BMI | n/a | n/a |
| (Geaney et al., 2016) Non-randomized trial | Reduction of saturated fat, sugar and salt, increase in fiber and FV, portion size control.  (*Environmental restructuring:* Opportunity;  *Restriction:* Opportunity) | Price discounts for whole fresh fruit. (*Incentivization:* Opportunity/ Motivation) | Strategic positioning of healthier alternatives. (*Environmental restructuring:* Opportunity) |  | Nutrition education comprised of monthly group nutrition presentations, detailed group nutrition information (daily and monthly posters, leaflets and emails) and individual nutrition consultations. Each participant attended three individual nutrition consultations (BL, F/U at 3–4 months and follow-up at 7-9 months.  *(Education:* Capability; *Persuasion*: Motivation) |  | n/a | ↓BMI, NS SBP, DBP, WC | ↓Fat, Sodium | n/a |
| (Ferdowsian et al., 2010) Non- randomized trial | Low-fat vegan options offered daily. (*Environmental restructuring:* Opportunity) |  |  |  | Group meetings with presentations, group discussion and cooking demonstration.  *(Education:* Capability; *Persuasion*: Motivation; *Modelling:* Motivation and Capability) |  | n/a | ↓Weight, BMI, WC NS LDL, HDL. | ↓%E Saturated fat, % E total fat, ↑Fiber | n/a |
| (Goetzel et al., 2010) Non- randomized trial | Changing cafeteria menus.  (*Environmental restructuring;* Opportunity) |  | Point of choice messages to encourage healthy eating and physical activity by strategically placing signs in front of cafeterias.  *(Persuasion*: Motivation) |  | Health promotion and risk reduction programs. Dissemination of health education materials; physical activity and weight management counselling.  *(Education:* Capability; *Persuasion:* Motivation) |  | n/a | ↓SBP, DBP, Cholesterol. Weight and BMI maintained in IG. | n/a | n/a |
| (Hjarnoe and Leppin, 2013) Pre/Post |  |  |  | Two-day course on healthy cooking for all chefs and staff with cooking responsibilities which was run over 5 alternate days.  *(Training:* Capability) |  |  | n/a | ↓Metabolic syndrome | ↓Intake of high sugar products | n/a |
| (Fernandez et al., 2015) RCT | Low sodium soup and reducing meals by 100 calories. (*Environmental restructuring:* Opportunity;  *Restriction:* Opportunity) | Half portions. FV sides subsidized using ‘Buy 3, Get 1 Free’ punch cards.  (*Incentivization:* Opportunity/ Motivation) | Healthy beverage signs. (*Persuasion:* Motivation) | Chef training workshop and a refresher lead by the dietitian on ways to cook healthier.  *(Training:* Capability) | Brochures on nutrition and physical activity. Educational posters and a website with wellness information.  *(Education:* Capability; *Persuasion:* Motivation) |  | n/a | ↓BMI, Weight | n/a | n/a |
| (Engbers et al., 2007) Non- randomized trial |  |  | Placement of informational sheets in close vicinity to food products. Every 4 weeks, 1 group out of 6 product groups was chosen and highlighted. Each group of food products was repeated once during the year. On the sheets the energy (kcal) value of 6 products was translated into the number of minutes needed to perform a certain activity to burn these calories.  (*Persuasion:* Motivation; *Environmental restructuring:*  Opportunity) |  |  |  | n/a | ↓Cholesterol, ↑HDL, SBP | n/a | n/a |
| (Mishra et al., 2013b) RCT | At intervention sites with cafeterias, food service managers were asked to include low-fat plant-based menu options, such as oatmeal, minestrone or lentil soup, veggie burgers and Portobello sandwiches, among the daily offerings.  *(Environmental restructuring:*  Opportunity) |  |  |  | Weekly lunch hour classes and group discussion following an established curriculum  (*Persuasion:* Motivation;  *Education:* Capability) |  | n/a | ↓Weight, BMI, Cholesterol, HBA1c, HDL, LDL | ↓%E total fat, %E saturated fat, Cholesterol ↑Fiber | n/a |
| (LaCaille et al., 2016) Non- randomized trial | Changes included reducing the size of serving spoons (BL) and offering half portions at half price. (*Environmental restructuring:* Opportunity;  *Restriction:* Opportunity) |  | Food items in the hospital cafeteria was labeled with calories, number of steps required to burn those calories, and with a traffic light color rating.  (*Persuasion:* Motivation;  *Environmental restructuring:* Opportunity) |  | Messages were offered through posters, table toppers, and a website in 3 phases. In the first phase, messages focused on educating employees about the meaning of the "traffic light" labels. The goal of the second phase was to educate employees about the meaning of energy balance and portion sizes. The final phase focused on underscoring the role of social support in losing and maintaining weight-loss.  *(Education:* Capability) |  | Null result | Null result: Weight, BM, WC | n/a | n/a |
| (Brehm et al., 2011) RCT | Taste tests with employees and researchers which lead to recommendations for improving the nutritional value of foods served in the cafeteria. Examples of recommendations included: (1) standardize and reduce portion sizes of entrees; (2) offer half portions of entrees; replace full-fat cheeses with reduced-fat cheeses on sandwiches and in recipes; (4) offer at least one healthier entrees on the menu; (5) offer a greater variety of fresh FV.  (*Environmental restructuring:* Opportunity;  *Restriction:* Opportunity) |  |  |  |  | Employee advisory committees and walking paths. (*Enablement*; Capability;  *Environmental restructuring:* Opportunity) | n/a | ↓Cholesterol, LDL, Triglyceride, Fasting glucose. NS BMI, body fat | ↓Saturated fat, Cholesterol | n/a |
| (Linde et al., 2012) RCT | Foods were classified as calorie smart for healthy portion sizes.  (*Environmental restructuring:* Opportunity; *Persuasion*: Motivation) |  |  |  | Posters and signs relating to healthy eating and exercise were placed in stairwells to enhance the stair environment. Other: physical activity was recorded with an infrared beam on staircases to record stair traffic. (*Persuasion:* Motivation;  *Education:* Capability;  *Environmental restructuring:* Opportunity;) |  | n/a | Null result: Weight | n/a | n/a |
| (Iriyama and Murayama, 2014) RCT | Provision of healthy cafeteria meals along with nutritional information defined as a meal containing 600–700kcal of energy and ≥120g of vegetables, with a fat/energy ratio of 20–25%) was served only to the IG at each worksite cafeteria (five days/week) for 6 months. The IG was instructed to consume these menus > 3 times per week.  (*Environmental restructuring:* Opportunity) |  |  |  | Health information was provided for 24 weeks using weekly nutrition notes placed on food trays and  one 20-minute individual counselling and a series of four 20-minute health education sessions in a small-group setting.  *(Education:* Capability) |  | n/a | ↓Weight, BMI | n/a | n/a |
| (Inoue et al., 2014) Non randomized trial | IG received a Japanese style lunch which provided balanced nutrition and sufficient vegetable consumption over the course of three months (600 kcal ≤ Energy < 650 kcal, Fat <18 g, Cholesterol ≤ 100 mg, Fiber ≥ 8 g, Total vegetables ≥130 g, Sodium chloride equivalent ≤ 3.8 g).  (*Environmental restructuring:* Opportunity; |  |  |  |  |  | ↑Vegetable | ↓Cholesterol, LDL cholesterol, SBP, DBP | ↓ Energy | n/a |
| (Levin et al., 2010) Non-randomized trial | IG cafeteria included low fat vegan menu options such as oatmeal, minestrone or lentil soup, veggie burgers and Portobello sandwiches. Approximately 1 breakfast item, and 4 lunch items (two entrees and side dishes) that met the diet guidelines were offered.  (*Environmental restructuring:* Opportunity; |  |  |  | Cooking demonstrations and nutrition education. *(Education:* Capability; *Modelling:* Motivation/  Capability) |  | n/a | ↓Weight, WC | ↓ Energy, Fat, Trans fat, Saturated fat, Cholesterol ↑ Fiber | n/a |
| (Mishra et al., 2013a) RCT | Participants at intervention sites were asked to follow a low-fat vegan diet consisting of whole grains, vegetables, legumes, and fruits, with no restriction on energy intake for 18 weeks. They were asked to avoid animal products and to minimize added oils, with a target of <3 g of fat per serving. They were also encouraged to favor foods with a low glycaemic index.  (*Environmental restructuring:* Opportunity;  *Restriction:* Opportunity) |  |  |  |  |  | n/a | n/a | ↓ %E fat, saturated fat, monosaturated fat ↓ Cholesterol ↑Fiber | n/a |
| (Lassen et al., 2011) RCT | Healthy canteen choices, free cold water, reduced soda and candy products.  *(Environmental restructuring:* Opportunity; *Incentivisation:* Opportunity/ Motivation) | Free fruit program.  *Incentivisation:* Opportunity/ Motivation) |  |  | Information and dialogue-based initiatives, food workshop/taste demonstrations, informational material (e.g. nutrition quizzes, dinner mats, computer-based activities, leaflets), monthly news magazine, healthy lunchtime clubs.  *(Education:* Capability) |  | ↑Fruit ↑FV | n/a | ↓%E fat, ↑Fiber | n/a |
| (Cook et al., 2001) Non- randomized trail | Inclusion of low-fat options and water as a beverage.  (*Environmental restructuring:* Opportunity) |  | Point of choice messages promoting FV.  (*Persuasion:* Motivation; *Environmental restructuring:* Opportunity) |  | Nutrition displays in the cafeteria.  *(Education:* Capability) |  | ↑Vegetable, NS Fruit | ↓ SBP, NS Weight, BMI, WC | ↓Fat score | n/a |
| (Geaney et al., 2010) Non- randomized trial | Reduction of food high in salt, fat and sugar. High-salt products and processed meat were replaced with low-salt options. Fresh herbs, spices and garlic were introduced to develop flavor. Salt was removed in all cooking. In the canteen, salt was removed from the tables, but salt sachets were available at service. No sauces or accompaniments were added to any meals without the customer’s consent. Cooking methods with oil were limited. Desserts were fruit base. (*Environmental restructuring:* Opportunity;  *Restriction:* Opportunity) | Staff members were encouraged to consume extra salad and vegetables options at no extra cost. (*Incentivization:* Opportunity/ Motivation) |  |  | Nutrition information on salt reduction and a healthy diet was displayed in the canteen area. *(Education:* Capability) |  | n/a | n/a | ↓Total sugar, Total fat, Saturated fat and Salt | n/a |
| (Emmons et al., 1999) RCT |  |  | At the point of purchase in cafeterias/vending machines the food labels met the Working Well Trial (WWT) criteria for fat or fiber.  (*Environmental restructuring:* Opportunity;  *Restriction:* Opportunity) |  |  | Catering policy to follow WWT guidelines. (*Enablement:* Capability/ Opportunity) | ↑FV | n/a | ↑Fiber | n/a |
| (Lassen et al., 2014) Non- randomized trail | For keyhole labelled meals, all recipes were modiﬁed and taste tests conducted to assess the acceptability of the modiﬁed foods. Food intake and edible plate waste measured through validated digital photographic method. The food was also weighed for intake estimation. (*Environmental restructuring:* Opportunity) | Fixed price was given to all menus at IG canteen and the CG canteen had buffet-by-weight meals.  (*Coercion*: Opportunity/Motivation) |  |  |  |  | ↑FV | n/a | ↓%E Fat, ↓Energy | n/a |
| (Lassen et al., 2012) Pre/Post | Fixed price of all menus at IG canteen and the CG canteen had buffet-by-weight meals.  *(Incentivization:* Opportunity/Motivation; *Environmental restructuring:* Opportunity) | CTA meals were offered twice weekly. Participants received CTA meals free of charge for themselves and for their families. *(Incentivization:* Opportunity/Motivation) |  |  |  |  | ↑Vegetable, ↑FV | n/a | ↓ Energy | n/a |
| (Lowe et al., 2010) RCT | Environmental change (i.e., the introduction of 10 new low-energy-density (ED).  *Environmental restructuring:* Opportunity | Pricing incentives for purchasing low-ED foods.  *(Incentivization:* Opportunity/Motivation) | Provision of labels for all foods sold at lunch, which listed ED, calories, and macronutrient content.  (*Persuasion:* Motivation; *Environmental restructuring:* Opportunity) |  | Education on low-ED eating delivered in four, 1-hour group session.  *(Education:* Capability) |  | ↑F | n/a |  | ↓ Energy content of lunch purchases |
| (Berkowitz et al., 2016) Time series | Consumption and plate waste data were collected for 5 weeks before and 7 weeks after introduction of 5 reduced-size entrées in a worksite lunch cafeteria. Full-size entrées were available throughout the entire study periods.  (*Environmental restructuring:* Opportunity; |  |  |  |  |  | n/a | n/a | ↓ Energy, ↓Total fat, ↓Cholesterol | Null result |
| (Vermeer et al., 2011) RCT | IG 1: smaller portion (2/3 the size of the existing portion) was offered in addition to the existing portion and proportional pricing. IG 2: smaller portion was added to the assortment and value size pricing (that is, a lower price per unit for large portions than for small portions).  (*Environmental restructuring:* Opportunity) |  |  |  |  |  | n/a | n/a | n/a | Null result |
| (Steenhuis et al., 2004) RCT | Increased availability of low-fat products and FV.  *Environmental restructuring:* Opportunity) |  | Attention was drawn to the new added products by placing a sign in front of them with the phrase ‘new and healthy’ on it. In the labelling program, low-fat products in 6 food product categories were labelled with a sign in front of the product.  *(Persuasion:* Motivation) |  | Education Program: Information was given to increase awareness, change attitudes, increasing self-efficacy, teaching skills and managing social influences.  *(Education:* Capability) |  | n/a | n/a | Null result | ↓Dessert sales with labelling program |
| (Thorndike et al., 2014) Time series |  |  | Phase 1 was a 3-month color-coded labeling intervention (red = unhealthy, yellow = less healthy, green = healthy). Phase 2 increased the visibility and convenience of some green items.  *Persuasion:* Motivation) |  |  |  | n/a | n/a | n/a | ↓Red sales items,↑ Green sales items |
| (Thorndike et al., 2012) Pre/Post |  |  | Phase 1 was a 3-month color-coded labeling intervention (red = unhealthy, yellow = less healthy, green = healthy). Phase 2 increased the visibility and convenience of some green items.  *(Persuasion:* Motivation) |  |  |  | n/a | n/a | n/a | ↓Red sales items, ↑ Green sales items |
| (Vyth et al., 2011) RCT |  |  | Choices logo was used to promote healthier eating for a 3-week period in IG cafeteria. Same sandwiches and soups were offered every day in addition to the Choices sandwich and soup. Choices logo was also placed on fresh fruit.  Duration: 9 weeks.  (*Persuasion:* Motivation;  *Environmental restructuring:* Opportunity) |  |  |  | n/a | n/a | n/a | Null result on sales of sandwiches, soups and salads, ↑ Fruit sales |
| (Kottke et al., 2013) Pre/Post |  | Reduced price of salad bar purchases by 50%. The subsidy was publicized through an e-mail to all employees and by a large poster in the cafeteria.  *(Incentivization:*Opportunity/Motivation; *Coercion*: Opportunity/Motivation) |  |  |  |  | n/a | n/a | n/a | ↑ Salad bar sales |
| (Levy et al., 2012) Pre/Post |  |  | The first intervention was a traffic light color-coded labeling system: healthy items (labeled green) and unhealthy items (labeled red). The second intervention manipulated “choice architecture” by physically rearranging certain cafeteria items, making green-labeled items more accessible.  (*Persuasion:* Motivation;  *Environmental restructuring:* Opportunity) |  |  |  | n/a | n/a | n/a | ↓ Red sales items, ↑ Green sales items |
| (Perlmutter et al., 1997) Pre/Post | 7 entrees from the cafeteria were modified to low total fat to <30% of energy and sodium to <1000 mg per serving and with nutrient information available. Modified standardized recipes and marketing of modified entrees was developed.  (*Environmental restructuring:* Opportunity) |  |  |  | Nutrient information was displayed on a large sign for all modified entrees being served that week.  (*Persuasion:* Motivation;  *Education:* Capability) |  | n/a | n/a | n/a | Null result |
| (Chen et al., 2017) Time series |  |  | Dissemination of information on traffic light labelling. Phase 2: implementation of the traffic light labelling in the buffet. The labeling included red (unhealthy/stop), yellow (moderately healthy/wait) and green traffic light labels (healthy/go).  (*Persuasion:* Motivation;  *Environmental restructuring:* Opportunity) |  |  |  | n/a | n/a | n/a | ↓Red sales items, ↑ Green sales items |
| (Levin, 1996) Non-randomized |  |  | Poster on low-fat entrée selection and heart shaped labels were placed next to 3 targeted entrees (bean burritos, potato and chili burritos and a turkey, lettuce and tomato sandwich) on the menu board.  (*Persuasion:* Motivation;  *Environmental restructuring:* Opportunity) |  |  |  | n/a | n/a | n/a | ↑Low-fat entrees sales |
| (van Kleef et al., 2012) Pre/Post |  |  | Each week an alternative snack arrangement was on display i.e. 25% healthy at top shelves, 25% healthy at bottom shelves, 75% healthy at top shelves, and 75% healthy at bottom shelves. All products were sold at €0.85 except for fresh fruits (i.e., apples, oranges and bananas) which were sold at €0.50. All four conditions of assortment structures were displayed for one week.  (*Environmental restructuring:* Opportunity) |  |  |  | n/a | n/a | n/a | ↑Healthy snacks sales |
| (Sonnenberg et al., 2013) Pre/Post |  |  | Traffic light labeling intervention in which all food and beverages served in the cafeteria were categorized by a negative or positive criterion.  (*Persuasion:* Motivation;  *Environmental restructuring:* Opportunity) |  | Dietitian support to answer questions about the labels and educate customers about the program. Pocket-sized pamphlets on labeling, calorie, fat, and saturated fat content of all items were supplied.  *(Education:* Capability) |  | n/a | n/a | n/a | ↑no of customers looked nutrition labelling, ↑Green sale items, ↓Red sales items |
| (Stites et al., 2015) Pre/Post |  | Participants were provided 20, $1.25 lunch vouchers for use in the 4-week full-intervention phase. *(Incentivisation:* Opportunity/Motivation) | The online pre-ordering system was designed to allow employees to order their lunches hours in advance of mealtime while viewing the nutrient content of the food choices. Daily and weekly specials rotated on a 4-week cyclic menu. (*Environmental restructuring:* Opportunity) |  | Mindful eating training was provided to participants. Topics included the definition of mindful eating, education on different types of hunger, and tips on how to be more mindful.  *(Education:* Capability;  *Enablement:* Capability/ Opportunity) |  | n/a | n/a | n/a | ↑in lunch meals sold with ↓ kilocalories and ↓ fat |
| (Wolfenden et al., 2015) RCT | Provided a total of 6 FV (such as fresh fruit, salads or salad sandwiches) and non-sugar-sweetened drink products for sale at their club canteen. Substitution of high fat/energy products with low fat/energy products and introduce other ‘healthier’ products for sale.  (*Environmental restructuring:* Opportunity;  *Restriction:* Opportunity) | Pricing strategies were encouraged to ensure that FV and non-sugar sweetened drink products were priced competitively compared to similar less healthy products. (*Coercion*: Opportunity/Motivation; *(Incentivisation:*Opportunity/Motivation) | Clubs were required to ensure at least 75% of non-alcoholic drinks in the canteen fridge were non-sugar-sweetened beverages and were positioned in the upper half of the fridge. Clubs were to ensure FV and non-sugar-sweetened drink products were displayed within view of consumers at all times. (*Environmental restructuring:* Opportunity) |  | Promotional strategies to improve the physical environment included encouraging FV and non-sugar-sweetened drink purchase via meal deals, signage and posters to draw customer’s attention to such products.  (*Persuasion:* Motivation) |  | n/a | n/a | n/a | ↑availability and sales of FV and non-sugar sweetened beverages |
| (Jeffery et al., 1994) Non- randomized trial | Doubling the number of fruit choices (6), increasing salad ingredient selections by 3.  (*Environmental restructuring:* Opportunity) | Reducing the price of fruit and salad by 50%.  (*Coercion*: Opportunity/Motivation) |  |  | Advertisements of intervention posted in cafeteria and through employees’ mailbox.  *(Education:* Capability; *Persuasion:* Motivation) |  | n/a | n/a | n/a | ↑salad and fruit sales |
| (Mazza et al., 2018) Time series | Oppositional pairing of less healthy food with a healthy alternative. (*Environmental restructuring:* Opportunity) | Soda price increase, water price decrease.  *Environmental restructuring:* Opportunity;  *Persuasion:* Motivation) | Traffic light labeling, emoticons and health messages, social norm messages and color grouping.  (*Persuasion:* Motivation;  *Environmental restructuring:* Opportunity) |  |  |  | n/a | n/a | n/a | ↑healthy beverage and chip sales with traffic light labelling in Phase 2  ↓healthy beverage sales (color grouping, social norms, oppositional pairing),  healthy chip sales with water price ↓ and soda price ↑ with traffic light labeling |
| (Viera et al., 2019) Pre/Post |  |  | One cafeteria received Physical Activity Calorie Expenditure (PACE ) labels which showed the calories in the food as well as an image of someone walking and the estimated number of miles needed to “burn off” the calories. The two other cafeterias received calorie-only labels. (*Persuasion:* Motivation;  *Environmental restructuring:* Opportunity) |  |  |  | n/a | n/a | n/a | ↓calorie content of foods purchased with PACE and calorie only labels |
| (Thorndike et al., 2019) Time series |  |  | Traffic light food labels and choice architecture (product placement) changes.  (*Persuasion:* Motivation;  *Environmental restructuring:* Opportunity) |  |  |  | n/a | n/a | n/a | ↓red-labeled items) ↑green-labeled items |
| (Pechey et al., 2019) RCT | Increasing the proportion of healthier (i.e. lower energy) cooked meals, snacks, cold drinks and sandwiches while decreasing the number of less health options. Healthier cooked meals (excluding breakfast) were defined as having under 300 kcal for a meal component typically served with an additional potato or rice side or under 500 kcal for a complete meal. Healthier sandwiches were defined as those under 350 kcal. Healthy snacks were defined as savory snacks under 120 kcal per pack, sweet snacks under 150 kcal per pack and cold drinks under 50 kcal per pack. (*Environmental restructuring:* Opportunity;  *Restriction:* Opportunity) |  |  |  |  |  | n/a | n/a | n/a | ↓energy foods purchased |
| (Hollands et al., 2018)  RCT | Reduce at least 10% the portion sizes of foods available in cafeterias from targeted categories (main meals, sides, desserts, cakes). (*Environmental restructuring:* Opportunity;  *Restriction:* Opportunity) |  |  |  |  |  | n/a | n/a | n/a | ↓energy purchased |
| (Vasiljevic et al., 2018) RCT |  |  | Labelling all cafeteria products for which such information was available with their calorie content (e.g. “250 Calories”) displayed in the same font style and size as for price. (*Environmental restructuring:* Opportunity;  *Persuasion:* Motivation) |  |  |  | n/a | n/a | n/a | No overall effect of the intervention. |
| (Vasiljevic et al., 2019) RCT |  |  | Calorie content was prominently displayed in bold capitalized Verdana typeface with a minimum font size of 14 e.g. 120 calories.  (*Environmental restructuring:* Opportunity; *Persuasion:* Motivation) |  |  |  | n/a | n/a | n/a | High levels of intervention acceptability: Null effect on daily energy purchased |

FV fruit and vegetable; RCT randomized control trial; n/a not assessed; WC waist circumference; SBP systolic blood pressure; Diastolic blood pressure; BMI body mass index; NS non-significant

**S6 File**

**Table 4 S6**. Review quality of studies.

| **Reference** | **Description** | | **Sampling** | | | **Measurement** | | | | | **Data Analysis** | | | | | **Interpretation of results** | | | **Total scoring** | **Quality rating** |
| --- | --- | --- | --- | --- | --- | --- | --- | --- | --- | --- | --- | --- | --- | --- | --- | --- | --- | --- | --- | --- |
|  | 1. | 2. | 1. | 2. | 3. | 1. | 2. | 3. | 4. | 5. | 1. | 2. | 3. | 4. | 5. | 1. | 2. | 3. |  |  |
| (Bandoni et al., 2011) | + | + | + | + | + | + | ? | ? | ? | + | + | + | + | + | ? | + | + | + | 14 | Fair |
| (Beresford et al., 2001) | + | + | + | + | + | + | + | + | + | + | + | + | + | + | + | - | + | + | 17 | Good |
| (Engbers et al., 2006) | + | + | + | + | + | + | - | - | + | ? | + | - | ? | + | + | ? | + | + | 12 | Limited |
| (Franco et al., 2013) | + | + | + | + | + | + | + | + | + | ? | + | + | + | ? | + | - | + | + | 15 | Fair |
| (Beresford et al., 2000) | + | + | + | + | + | + | + | + | + | + | + | + | + | + | + | - | + | + | 17 | Good |
| (Thorsen et al., 2010) | - | + | - | - | + | + | - | - | + | ? | + | + | ? | ? | ? | - | - | - | 6 | Limited |
| (Buller et al., 1999) | + | + | + | + | + | + | - | - | + | ? | + | + | ? | ? | ? | ? | ? | + | 10 | Limited |
| (Kushida and Murayama, 2014) | + | + | + | + | + | + | - | - | + | ? | + | + | ? | + | + | - | ? | ? | 11 | Limited |
| (Uglem et al., 2013) | + | + | + | + | + | + | - | - | + | ? | + | + | ? | + | + | - | ? | ? | 11 | Limited |
| (Leighton et al., 2009) | + | + | + | + | + | + | - | - | + | ? | + | + | ? | + | ? | + | - | - | 11 | Limited |
| (Thorsteinsson et al., 1994) | + | + | - | + | + | + | - | - | - | ? | + | + | - | - | - | ? | - | ? | 7 | Limited |
| (Geaney et al., 2016) | + | + | + | + | + | + | + | ? | + | ? | + | + | + | + | + | - | + | + | 15 | Fair |
| (Ferdowsian et al., 2010) | + | + | + | + | + | + | - | + | + | + | + | + | + | + | - | ? | + | + | 15 | Fair |
| (Goetzel et al., 2010)(32) 2010 | + | + | + | + | + | + | + | + | + | + | + | + | + | + | + | - | + | + | 17 | Good |
| (Hjarnoe and Leppin, 2013) | + | + | + | + | + | + | - | - | + | ? | + | + | ? | + | ? | - | + | + | 12 | Limited |
| (Fernandez et al., 2015) | + | + | + | + | + | + | ? | ? | + | ? | + | + | + | + | + | - | - | - | 12 | Limited |
| (Engbers et al., 2007) | + | + | + | + | + | + | - | - | + | ? | + | + | ? | + | + | - | + | + | 13 | Limited |
| (Mishra et al., 2013b) | + | + | + | + | - | + | - | ? | ? | ? | + | + | + | + | + | ? | + | + | 12 | Limited |
| (LaCaille et al., 2016) | + | + | + | + | + | + | + | ? | + | ? | + | + | + | + | + | - | - | + | 14 | Fair |
| (Brehm et al., 2011) | + | + | + | + | + | + | - | - | + | ? | + | + | + | + | + | - | + | + | 14 | Fair |
| (Linde et al., 2012) | + | + | + | + | + | + | + | ? | ? | - | - | + | + | + | + | + | + | + | 14 | Fair |
| (Iriyama and Murayama, 2014) | + | + | + | + | + | + | ? | ? | + | ? | + | + | + | + | ? | - | ? | + | 12 | Limited |
| (Inoue et al., 2014) | + | + | - | + | - | + | - | - | + | ? | + | + | ? | + | ? | ? | - | - | 8 | Limited |
| (Levin et al., 2010) | + | + | + | + | - | + | - | - | ? | ? | + | + | + | ? | ? | + | - | + | 10 | Limited |
| (Mishra et al., 2013a) | + | + | + | + | - | + | ? | ? | ? | ? | + | + | + | + | + | ? | + | + | 12 | Limited |
| (Lassen et al., 2011) | + | + | + | + | + | + | - | - | + | ? | + | + | ? | + | ? | - | - | - | 10 | Limited |
| (Cook et al., 2001) | + | + | + | + | + | + | - | - | + | - | + | + | + | + | - | + | + | + | 14 | Fair |
| (Geaney et al., 2010) | + | + | + | + | + | + | - | - | + | ? | + | + | + | + | ? | ? | ? | ? | 11 | Limited |
| (Emmons et al., 1999) | + | + | + | + | + | + | ? | ? | + | ? | + | + | + | + | + | _ | ? | + | 13 | Limited |
| (Lassen et al., 2014) | + | + | + | + | - | + | + | ? | + | ? | + | + | ? | ? | ? | - | + | + | 11 | Limited |
| (Lassen et al., 2012) | + | + | + | + | + | + | + | ? | + | ? | + | + | + | + | ? | - | + | + | 14 | Fair |
| (Lowe et al., 2010) | + | + | + | + | + | + | ? | ? | + | ? | + | + | + | + | ? | - | + | + | 13 | Limited |
| (Berkowitz et al., 2016) | + | + | + | - | + | + | - | - | + | - | + | + | - | - | - | - | - | - | 8 | Limited |
| (Vermeer et al., 2011) | + | + | + | + | + | + | + | + | ? | - | + | + | + | + | + | - | - | + | 14 | Fair |
| (Steenhuis et al., 2004) | + | + | + | + | + | + | + | ? | + | + | + | + | + | + | + | + | + | + | 17 | Good |
| (Thorndike et al., 2014) | + | + | + | + | + | + | ? | ? | + | ? | + | + | + | - | ? | - | ? | ? | 10 | Limited |
| (Thorndike et al., 2012) | - | - | - | - | - | + | ? | ? | + | + | + | + | + | ? | ? | - | - | ? | 6 | Limited |
| (Vyth et al., 2011) | + | + | + | + | - | + | ? | ? | ? | ? | + | + | + | ? | ? | - | ? | ? | 8 | Limited |
| (Kottke et al., 2013) | - | + | - | + | - | + | - | - | + | ? | + | - | - | - | - | - | - | - | 5 | Limited |
| (Levy et al., 2012) | - | + | + | + | - | + | - | - | + | ? | + | + | + | ? | ? | - | - | - | 7 | Limited |
| (Perlmutter et al., 1997) | - | + | - | + | + | + | - | - | + | ? | + | + | - | - | - | - | - | - | 7 | Limited |
| (Chen et al., 2017) | - | + | + | - | + | + | - | ? | + | ? | + | + | ? | ? | ? | - | - | - | 7 | Limited |
| (Levin, 1996) | + | + | - | - | - | + | - | - | + | ? | + | + | ? | ? | ? | - | ? | - | 6 | Limited |
| (van Kleef et al., 2012) | - | + | - | - | + | + | - | - | + | ? | + | + | ? | ? | ? | - | - | - | 6 | Limited |
| (Sonnenberg et al., 2013) | - | + | - | - | + | + | - | - | + | ? | + | + | ? | ? | - | - | - | - | 6 | Limited |
| (Stites et al., 2015) | + | + | + | + | + | + | - | - | + | ? | + | + | ? | ? | ? | - | - | - | 9 | Limited |
| (Wolfenden et al., 2015) | + | + | + | + | + | + | ? | ? | ? | ? | + | + | ? | - | ? | - | + | ? | 9 | Limited |
| (Jeffery et al., 1994) | - | + | + | - | - | + | ? | ? | + | ? | + | + | ? | + | - | - | - | - | 7 | Limited |
| (Mazza et al., 2018) | + | + | + | + | + | + | + | - | + | ? | + | + | + | ? | + | ? | + | - | 13 | Limited |
| (Viera et al., 2019) | + | + | + | + | - | + | + | - | + | ? | + | + | + | + | + | - | + | + | 14 | Fair |
| (Thorndike et al., 2019) | + | + | + | + | + | + | + | - | + | ? | + | + | + | + | + | - | + | + | 15 | Fair |
| (Pechey et al., 2019) | + | + | + | + | + | + | + | - | + | - | + | + | + | + | + | + | + | + | 16 | Fair |
| (Hollands et al., 2018) | + | + | + | + | - | + | + | - | + | + | + | + | + | + | + | - | + | + | 15 | Fair |
| (Vasiljevic et al., 2018) | + | + | + | + | + | + | + | + | + | + | + | + | + | + | + | + | + | - | 17 | Good |
| (Vasiljevic et al., 2019) | + | + | + | + | + | + | + | + | + | + | + | + | + | + | + | + | + | - | 17 | Good |

+ Yes; - No; ? Unclear

Good: Studies with none or one limitation; Fair: Studies with 2-4 limitations; Limited execution: Studies with 5 or more limitations
